# Extensive alterations of the whole-blood transcriptome are associated with body mass index: results of an mRNA profiling study involving two large population-based cohorts

**DOI:** 10.1186/s12920-015-0141-x

**Published:** 2015-10-15

**Authors:** Georg Homuth, Simone Wahl, Christian Müller, Claudia Schurmann, Ulrike Mäder, Stefan Blankenberg, Maren Carstensen, Marcus Dörr, Karlhans Endlich, Christian Englbrecht, Stephan B. Felix, Christian Gieger, Harald Grallert, Christian Herder, Thomas Illig, Jochen Kruppa, Carola S. Marzi, Julia Mayerle, Thomas Meitinger, Andres Metspalu, Matthias Nauck, Annette Peters, Wolfgang Rathmann, Eva Reinmaa, Rainer Rettig, Michael Roden, Arne Schillert, Katharina Schramm, Leif Steil, Konstantin Strauch, Alexander Teumer, Henry Völzke, Henri Wallaschofski, Philipp S. Wild, Andreas Ziegler, Uwe Völker, Holger Prokisch, Tanja Zeller

**Affiliations:** Interfaculty Institute for Genetics and Functional Genomics, University Medicine and Ernst-Moritz-Arndt-University Greifswald, Greifswald, Germany; Research Unit Molecular Epidemiology, Helmholtz Zentrum München, German Research Center for Environmental Health, Neuherberg, Germany; German Center for Diabetes Research (DZD), Neuherberg, Germany; Institute of Epidemiology II, Helmholtz Zentrum München, German Research Center for Environmental Health, Neuherberg, Germany; Klinik für Allgemeine und Interventionelle Kardiologie, Universitäres Herzzentrum Hamburg, Hamburg, Germany; DZHK (German Centre for Cardiovascular Research), partner site Hamburg/Kiel/Lübeck, Hamburg, Germany; DZHK (German Centre for Cardiovascular Research), partner site Greifswald, Greifswald, Germany; Present Address: The Charles Bronfman Institute for Personalized Medicine, Genetics of Obesity & Related Metabolic Traits Program, Icahn School of Medicine at Mount Sinai, New York, USA; Institute for Clinical Diabetology, German Diabetes Center, Leibniz Center for Diabetes Research at Heinrich Heine University Düsseldorf, Düsseldorf, Germany; German Center for Diabetes Research (DZD e.V.), partner site Düsseldorf, Düsseldorf, Germany; Department of Internal Medicine B, University Medicine Greifswald, Greifswald, Germany; Institute of Anatomy and Cell Biology, University Medicine Greifswald, Greifswald, Germany; Hannover Unified Biobank, Hannover Medical School, Hannover, Germany; Institute for Human Genetics, Hannover Medical School, Hannover, Germany; Institut für Medizinische Biometrie und Statistik, Universität zu Lübeck, Universitätsklinikum Schleswig-Holstein, Campus Lübeck, Lübeck, Germany; Department of Internal Medicine A, University Medicine Greifswald, Greifswald, Germany; Institute of Human Genetics, Helmholtz Zentrum München, German Research Center for Environmental Health, Neuherberg, Germany; DZHK (German Centre for Cardiovascular Research), partner site Munich, Munich, Germany; Institut für Humangenetik, Technische Universität München, München, Germany; Munich Heart Alliance, Munich, Germany; Estonian Genome Center, University of Tartu, Tartu, Estonia; Institute of Clinical Chemistry and Laboratory Medicine, University Medicine Greifswald, Greifswald, Germany; Institute of Biometrics and Epidemiology, German Diabetes Center, Leibniz Center for Diabetes Research at Heinrich Heine University Düsseldorf, University Düsseldorf, Düsseldorf, Germany; Institute of Molecular and Cell Biology, University of Tartu, Tartu, Estonia; Institute of Physiology, University Medicine Greifswald, Karlsburg, Germany; Division of Endocrinology and Diabetology, University Hospital Düsseldorf, Düsseldorf, Germany; Institute of Genetic Epidemiology, Helmholtz Zentrum München, German Research Center for Environmental Health, Neuherberg, Germany; Institute of Medical Informatics, Biometry and Epidemiology, Chair of Genetic Epidemiology, Ludwig-Maximilians-Universität, Munich, Germany; Institute for Community Medicine, University Medicine Greifswald, Greifswald, Germany; Preventive Cardiology and Preventive Medicine, Department of Medicine 2, University Medical Center of the Johannes Gutenberg-University Mainz, Mainz, Germany; Center for Thrombosis and Hemostasis, University Medical Center of the Johannes Gutenberg-University Mainz, Mainz, Germany; DZHK (German Centre for Cardiovascular Research), partner site Rhine-Main, Mainz, Germany; Zentrum für Klinische Studien, Universität zu Lübeck, Lübeck, Germany; School of Statistics, Mathematics and Computer Science, University of KwaZulu-Natal, Pietermaritzburg, South Africa

**Keywords:** Transcriptomics, Transcriptome-wide association study (TWAS), BMI, Obesity, Insulin resistance, Type 2 diabetes, Oxidative stress, Insulin signaling

## Abstract

**Background:**

Obesity, defined as pathologically increased body mass index (BMI), is strongly related to an increased risk for numerous common cardiovascular and metabolic diseases. It is particularly associated with insulin resistance, hyperglycemia, and systemic oxidative stress and represents the most important risk factor for type 2 diabetes (T2D). However, the pathophysiological mechanisms underlying these associations are still not completely understood. Therefore, in order to identify potentially disease-relevant BMI-associated gene expression signatures, a transcriptome-wide association study (TWAS) on BMI was performed.

**Methods:**

Whole-blood mRNA levels determined by array-based transcriptional profiling were correlated with BMI in two large independent population-based cohort studies (KORA F4 and SHIP-TREND) comprising a total of 1977 individuals.

**Results:**

Extensive alterations of the whole-blood transcriptome were associated with BMI: More than 3500 transcripts exhibited significant positive or negative BMI-correlation. Three major whole-blood gene expression signatures associated with increased BMI were identified. The three signatures suggested: i) a ratio shift from mature erythrocytes towards reticulocytes, ii) decreased expression of several genes essentially involved in the transmission and amplification of the insulin signal, and iii) reduced expression of several key genes involved in the defence against reactive oxygen species (ROS).

**Conclusions:**

Whereas the first signature confirms published results, the other two provide possible mechanistic explanations for well-known epidemiological findings under conditions of increased BMI, namely attenuated insulin signaling and increased oxidative stress. The putatively causative BMI-dependent down-regulation of the expression of numerous genes on the mRNA level represents a novel finding. BMI-associated negative transcriptional regulation of insulin signaling and oxidative stress management provide new insights into the pathogenesis of metabolic syndrome and T2D.

**Electronic supplementary material:**

The online version of this article (doi:10.1186/s12920-015-0141-x) contains supplementary material, which is available to authorized users.

## Background

The past two decades saw obesity surpass malnutrition and infectious diseases as the greatest contributors to morbidity and mortality. This obesity epidemic is also driving the world-wide endemic development of type 2 diabetes (T2D) [1]. Body mass index, the most commonly used anthropometric measure in the context of health risks related to obesity, is correlated with metabolic disorders, cardiovascular and all-cause mortality [2].

Regulation of body weight is complex and affected by genetic as well as environmental factors. Heritability of anthropometric measures such as BMI is as high as 40 to 70 % [3]. This prompted intense research for underlying genetic factors to unravel the metabolic networks controlling body mass [4]. Monogenic mutations such as those described for *LEP*, *LEPR*, *POMC*, and *MC4R* cause only a minority of obesity cases whereas in most individuals obesity is based on a polygenic predisposition amplified by an obesogenic Western lifestyle [4].

Genome-wide association studies (GWAS) have detected a large number of genes modulating levels of and susceptibility to adiposity. However, the effect sizes of these common variants are small, with a limited predictive value for obesity risk [5]. Gene expression studies have emerged as a promising approach for the analysis of gene regulatory networks and might allow the identification of pathways linked to body mass regulation [6]. Previous studies on obese subjects demonstrated that blood cells represent a robust model to study the maintenance of energy homeostasis and its interaction with body weight [7].

In order to identify potentially disease-relevant BMI-related gene expression signatures, we determined whole-blood mRNA levels of 1977 individuals from two large independent population-based cohort studies (Kooperative Gesundheitsforschung in der Region Augsburg (KORA) and Study of Health in Pomerania (SHIP-TREND)) within the frame of the DZHK MetaXpress consortium by array-based transcriptional profiling and subsequently correlated mRNA abundances and BMI in the current study. In addition to the demonstration of extensive BMI-associated alterations of the whole-blood transcriptome, three distinct gene expression signatures associated with increased BMI were detected. The observation of BMI-associated decreased expression of genes involved in insulin signaling and protection against oxidative stress provides new insight into the pathomechanisms underlying obesity-mediated insulin resistance and systemic oxidative stress contributing to the development of type 2 diabetes (T2D).

## Methods

### Study populations

All subjects were of European ancestry. Written, informed consent has been obtained from participants and the studies were approved by the Ethics Committees of the University of Greifswald and the Bavarian Medical Association for SHIP and KORA, respectively.

#### KORA F4

The KORA (Cooperative Health Research in the Region of Augsburg) F4 survey has been described before [8]. Briefly, 1653 individuals aged 55 to 74 years from the city Augsburg in the southeast of Germany and two adjacent counties participated in the population-based KORA survey S4 that was conducted between 1999 and 2001 [8]. This cohort was reinvestigated in the KORA survey F4 in 2006–2008. Study design, sampling method and data collection have been described in detail elsewhere [9, 10]. The study presented here is based on a KORA F4 subsample of 988 individuals aged 62 to 81 years.

#### SHIP-TREND

The Study of Health in Pomerania (SHIP) is a longitudinal population-based cohort study in West Pomerania, a region in the northeast of Germany, assessing the prevalence and incidence of common population-relevant diseases and their risk factors. Baseline examinations for SHIP-TREND were carried out between 2008 and 2012, comprising 4,420 participants. Study design and sampling methods were previously described [11]. The present project is based on a subset of 989 individuals aged 20 to 81 years of the SHIP-TREND study population.

### Anthropometric measurements

Weight and height were measured using standard protocols as described elsewhere [8]. For the anthropometric measurements, calibrated, digital scales (Seca 862, Seca Germany) and a measuring stick (Seca 220, Seca, Germany) were used. The body mass index (BMI) was calculated as weight in kilograms divided by height in square meters.

### Preparation and quality control of whole-blood RNA and transcriptome analysis

Blood sample collection as well as RNA preparation were described in detail elsewhere [12]. Briefly, whole-blood samples were collected from participants of both studies between 8:00 a.m. and noon after overnight fasting and stored in PAXgene Blood RNA Tubes (BD). Subsequently, RNA was prepared using the PAXgene™ Blood miRNA Kit (QIAGEN, Hilden, Germany). Purity and concentration of RNA were determined using a NanoDrop ND-1000 UV–vis Spectrophotometer (Thermo Scientific). To ensure a constantly high quality of the RNA preparations, all samples were analyzed using RNA 6000 Nano LabChips (Agilent Technologies, Germany) on a 2100 Bioanalyzer (Agilent Technologies, Germany) according to the manufacturer’s instructions. Samples exhibiting an RNA integrity number (RIN) less than seven were excluded from further analysis. The Illumina TotalPrep-96 RNA Amplification Kit (Ambion, Darmstadt, Germany) was used for reverse transcription of 500 ng RNA into double-stranded (ds) cDNA and subsequent synthesis of biotin-UTP-labeled antisense-cRNA using this cDNA as the template. Finally, in total 3,000 ng of cRNA were hybridized with a single array on the Illumina HumanHT-12 v3 BeadChips, followed by washing and detection steps in accordance with the Illumina protocol. BeadChips were scanned using the Illumina Bead Array Reader.

The Illumina software GenomeStudio V 2010.1 was used to read the generated raw data, for imputation of missing values and sample quality control. Subsequently, raw gene expression data were exported to the statistical environment R, version 2.14.2 (R Development Core Team 2011). Data were normalized using quantile normalization and log_2_-transformation using the lumi 2.8.0 package from the Bioconductor open source software (http://www.bioconductor.org/).

### Gene expression data analysis

Individuals with missing BMI values or at least one of the covariates values (KORA F4: *n* = 5, SHIP-TREND: *n* = 0) were excluded from the analysis. In linear regression models, gene expression levels were regressed on BMI with adjustment for age, sex, red blood cell count (RBC), white blood cell count (WBC), hematocrit, platelet count, RNA quality (RIN), plate layout after RNA amplification (96 well plates), and sample storage time (time between blood donation and RNA preparation) [12]. It has been demonstrated earlier that KORA F4 and SHIP-TREND are comparable and can be meta-analyzed, even if the two cohorts differ in some minor aspects [12]. A sample size-weighted z-score based meta-analysis was used to combine the gene expression data from both cohorts (*n* = 1977) using the *metafor* meta-analysis package for R, version 1.4-0 (http://www.jstatsoft.org/v36/i03/). In order to test if any detected associations between BMI and whole-blood mRNA levels were mediated by BMI-dependent shifts in the proportions of different blood cell sub-types, an additional analysis with adjustment for relative lymphocyte, neutrophil, basophil, eosinophil and monocyte count was performed in SHIP-TREND, where these parameters were available for all individuals. Adjusting for these parameters did not substantially change the obtained results. The corresponding R^2^-values for effect size (beta), standard error, and -log_10_ p-value of the meta-analysis were 94, 99, and 91 %, respectively. The effect of an additional adjustment for homeostasis model assessment – insulin resistance (HOMA-IR) on the results was negligible. The corresponding R^2^-values for effect size (beta), standard error, and -log_10_ p-value of the meta-analysis were 99.56, 99.98, and 99.39 %, respectively. Only the 12,778 transcripts with a detection rate (defined as the proportion of observations with detection p-value < 0.05) above 50 % in both cohorts were considered. The Benjamini and Hochberg false discovery rate (FDR) was used to correct for multiple testing. Associations with an FDR < 0.01 were considered statistically significant. Pathway analyses were performed based on all significant gene-specific probes using the Ingenuity Pathway Analysis (IPA) software tool (IPA build version: 338830 M, content version: 23814503, release date: 2015-03-22; analysis Date: 2015-06-19; http://www.ingenuity.com/). The database underlying IPA is referred to as the Ingenuity Knowledge Base. Based on the Illumina ProbeIDs, the IPA software allocates probes to annotated genes and to corresponding objects in the Ingenuity Knowledge Base. The reference set was restricted to genes represented on the IlluminaHT-12 v3 BeadChip, and only human annotations were considered. In case multiple probes mapped to one gene, the probe exhibiting the smallest p-value was considered for downstream analyses. Pathway analyses were performed with IPA’s Core Analysis module. Overrepresentations of BMI-associated genes in functional categories and canonical pathways were calculated using a right-tailed Fisher’s exact test with a significance level of 0.05 after Benjamini and Hochberg correction. The resulting p-value identifies over-representation of input genes in a given process. Gene enrichment in canonical pathways is described by the ratio of the number of input genes that map to the pathway divided by the total number of genes in this pathway. Permutation analysis, which is described in detail in Additional file 1: Supplemental Materials, was performed for evaluating robustness of Ingenuity Pathway Analysis (IPA) and identifying potentially false positive over-representations.

For an overview of the number of significant and annotated probes as well as genes, see Additional file 2: Figure S1–S34. The detailed workflow for the analysis of Illumina gene expression microarray data was described recently within the MetaXpress consortium [12].

## Results and discussion

### General results

**Fig. 1:**
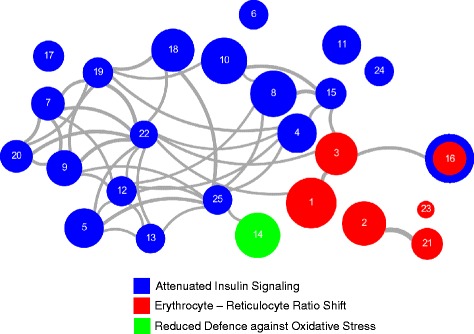
Overlap graph of the top 25 enriched IPA-pathways. Each node represents one pathway significantly enriched with BMI-associated gene-specific transcripts. The node size is proportional to the number of BMI-associated transcripts within each respective pathway. Edge shade and width refer to the overlap between pathways in terms of shared transcripts and were calculated based on the Jaccard similarity coefficient, which is defined as the size of the intersection divided by the size of the union of two sample sets. Edges between nodes are shown if transcripts are shared between pathways and if the Jaccard coefficient ≥ 90th percentile. Nodes, i.e., pathways are colored according to the three defined gene expression signatures. The extensive connectivity, particularly for the attenuated insulin signaling, represents the strong overlap among the pathways. Pathways are numbered according to their enrichment p-values. 1: EIF2 Signaling, 2: Mitochondrial Dysfunction, 3: Regulation of eIF4 and p70S6K Signaling, 4: PI3K/AKT Signaling, 5: Insulin Receptor Signaling, 6: Hypoxia Signaling in the Cardiovascular System, 7: Chronic Myeloid Leukemia Signaling, 8: Production of Nitric Oxide and Reactive Oxygen Species in Macrophages, 9: Pancreatic Adenocarcinoma Signaling, 10: ILK Signaling, 11: AMPK Signaling, 12: JAK/Stat Signaling, 13: Renal Cell Carcinoma Signaling, 14: NRF2-mediated Oxidative Stress Response, 15: Ceramide Signaling, 16: mTOR Signaling, 17: CTLA4 Signaling in Cytotoxic T Lymphocytes, 18: B Cell Receptor Signaling, 19: Prostate Cancer Signaling, 20: Glioma Signaling, 21: Oxidative Phosphorylation, 22: Non-Small Cell Lung Cancer Signaling, 23: Heme Biosynthesis II, 24: Cyclins and Cell Cycle Regulation, 25: PDGF Signaling

Within the MetaXpress Consortium of the German Center for Cardiovascular Research (DZHK), whole-blood mRNA levels of 1977 participants of the two independent population-based studies SHIP-TREND and KORA F4 (Table 1) were determined by array-based transcriptional profiling. Subsequently, whole-blood mRNA levels were associated with the phenotype BMI. Meta-analysis of the whole-blood gene expression data resulted in the identification of 3762 annotated genes whose transcript levels were significantly associated with BMI after adjusting for multiple testing [Benjamini and Hochberg false discovery rate (FDR) < 0.01] (Additional file 2: Table S1). Recently, the Data-driven Expression Prioritized Integration for Complex Traits (DEPICT) method was used to prioritize BMI-related genes [5]. Of the 989 genes prioritized by DEPICT as BMI-related, 388 exhibited significant mRNA levels in our analysis. For 151 of these transcripts, we could confirm association of the their expression levels with BMI (Additional file 2: Table S33). Of the 3762 genes described above, the correlation between mRNA abundance and BMI was positive for 1269 genes (33.7 %) and negative for 2411 genes (64.1 %), while 82 genes (2.2 %) exhibited inconsistent effect directions on the probe level. In the latter case, the different probes target different exons of the corresponding genes, indicating that individual mRNA isoforms specified by the same genes were inversely associated with BMI. Adjustment for HOMA-IR resulted in the detection of 1836 and 2602 transcripts exhibiting positive and negative BMI-correlations, respectively, with an overlap of 90.2 % and 94.1 % to the non-HOMA-IR-adjusted analysis, demonstrating that associations of BMI with transcript levels were largely independent of HOMA-IR (Additional file 2: Table S34). Using Ingenuity Pathways Analysis (IPA) software, we identified 128 and 63 canonical pathways exhibiting significant association with BMI after controlling the FDR at 0.05 and 0.01, respectively (Additional file 2: Table S5). In order to validate robustness across platforms, the pathway over-representation analysis was repeated using the web-based toolkit *WebGestalt* (bioinfo.vanderbilt.edu/webgestalt/) and the online database resources *KEGG* (genome.jp/kegg/) and *WikiPathways* (wikipathways.org). The significantly enriched pathways were essentially concordant with those detected using IPA (Additional files 3 and 4). Subsequent closer inspection of the assigned gene-specific transcripts revealed a strong overlap between the gene/protein content of numerous of the canonical IPA-pathways. The extensive connectivity of the nodes displayed in Fig. 1 represents this strong overlap among the pathways, particularly for the attenuated insulin signaling. We therefore restricted the interpretation of the pathway analysis to the top 25 IPA-pathways exhibiting the most significant associations and defined three prominent whole-blood gene expression signatures (see below) as the major outcome of the analysis.

**Table 1:** Cohort characteristics

Variables	SHIP-TREND	KORA F4
Mean age [years]	50.1 ± 13.7	70.4 ± 5.4
Females	555 (56.0 %)	493 (49.6 %)
Active smokers	214 (21.2 %)	66 (6.7 %)
Mean body height [cm]	169.8 ± 9.0	165.3 ± 8.8
Body weight [kg]	79.0 ± 15.1	78.9 ± 13.7
Body mass index (BMI) [kg/m^2^]	27.3 ± 4.6	28.9 ± 4.5
Waist circumference [cm]	88.0 ± 12.9	98.6 ± 12.1
Waist-to-height ratio (WHtR)	0.87 ± 0.09	0.91 ± 0.08
Fasting glucose [mg/dL]	97.9 ± 11.2	104.8 ± 22.8
2-hour glucose OGTT [mg/dL]	117.3 ± 36.7	128.7 ± 42.4
Fasting insulin [mU/L]	6.7 ± 8.3	10.4 ± 33.5
Homeostasis model assessment insulin resistance (HOMA-IR)	1.15 ± 1.72	2.82 ± 8.95
HbA1c [%]	5.19 ± 0.57	5.77 ± 0.69
Leptin [ng/mL]	15.6 ± 14.5	22.4 ± 24.3
Red blood cell count [Tpt/L]	4.63 ± 0.39	4.50 ± 0.40
White blood cell count [pt/nl]	5.72 ± 1.48	6.00 ± 1.81
Platelet count [pt/nl]	225.7 ± 50.3	244.7 ± 65.1
Hematocrit [l/l]	0.42 ± 0.03	0.41 ± 0.03
Systolic blood pressure (SBP) [mmHg]	124.4 ± 16.9	128.7 ± 20.0
Diastolic blood pressure (DBP) [mmHg]	76.6 ± 9.8	74.0 ± 10.1

Data are given as mean ± standard deviation or as number and percent (in parentheses). *OGTT* oral glucose tolerance test, *Tpt/L* 10^12^ cells/L

**Fig. 2:**
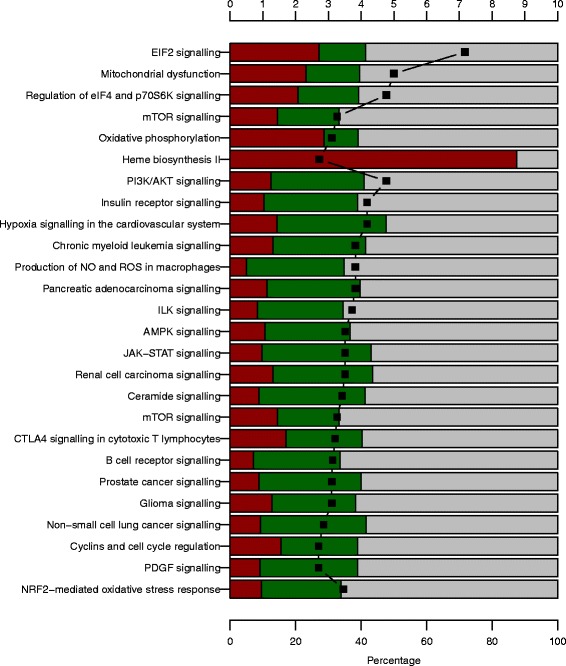
Ingenuity pathway analysis (IPA) results. The results are shown for the 25 most significantly enriched BMI-associated canonical IPA pathways. The percentage of transcripts that are positively, negatively or not significantly associated with BMI in the meta-analysis is provided as red, green and grey bars, respectively. Black squares display the –log_10_ p-value

The 25 pathways included *EIF2 Signaling*, *Mitochondrial Dysfunction*, *Regulation of eIF4 and p70S6K Signaling*, *PI3K/AKT Signaling*, *Insulin Receptor Signaling*, *Hypoxia Signaling in the Cardiovascular System*, *Chronic Myeloid Leukemia Signaling*, *Production of Nitric Oxide and Reactive Oxygen Species in Macrophages*, *Pancreatic Adenocarcinoma Signaling*, *ILK Signaling*, *AMPK Signaling*, *JAK/Stat Signaling*, *Renal Cell Carcinoma Signaling*, *NRF2-mediated Oxidative Stress Response*, *Ceramide Signaling*, *mTOR Signaling*, *CTLA4 Signaling in Cytotoxic T Lymphocytes*, *B Cell Receptor Signaling*, *Prostate Cancer Signaling*, *Glioma Signaling*, *Oxidative Phosphorylation*, *Non-Small Cell Lung Cancer Signaling*, *Heme Biosynthesis II*, *Cyclins and Cell Cycle Regulation*, and *PDGF Signaling* (Fig. 2 and Table 2). These 25 pathways comprised 396 genes whose transcript abundance in whole-blood was significantly associated with BMI (Additional file 2: Tables S4–S30). Overall, the correlations were positive for 171 genes (43.2 %) and negative for 225 genes (56.8 %). Permutation analysis confirmed over-representation of genes in all pathways but *PDGF Signaling* (for details see Additional file 1: Supplementary Materials).

**Table 2:** The 25 IPA-pathways most significantly enriched for BMI-associated gene-specific transcripts in the SHIP-TREND/KORA F4 meta-analysis

Defined gene expression signatures with assigned canonical IPA-pathways	FDR for genes with BMI-associated mRNA levels (all genes/ positively associated/ negatively associated)	Number of genes with BMI-associated mRNA levels (all genes/ positively associated/ negatively associated)	FDR from permutation analysis
Erythrocyte-Reticulocyte Ratio Shift			
EIF2 signaling	**6.8 × 10** ^**-8**^/ **6.3 × 10** ^**-14**^/ 5.8 × 10^-1^	70/ 46/ 24	0.0002
Mitochondrial dysfunction	**1.0 × 10** ^**-5**^/ **3.6 × 10** ^**-8**^/ 3.2 × 10^-1^	58/ 34/ 24	0.0002
Regulation of eIF4 and p70S6K signaling	**1.7 × 10** ^**-5**^/ **4.5 × 10** ^**-6**^/ 1.3 × 10^-1^	55/ 29/ 26	0.0002
mTOR signaling	**5.4 × 10** ^**-4**^/ 1.1 × 10^-2^/ 6.9 × 10^-2^	60/ 26/ 34	0.0059
Oxidative phosphorylation	**7.9 × 10** ^**-4**^/ **4.9 × 10** ^**-8**^/ N. A.	34/ 25/ 9	0.0002
Heme biosynthesis II	**1.9 × 10** ^**-3**^/ **4.5 × 10** ^**-6**^/ N. A.	7/ 7/ 0	0.0002
Attenuated Insulin Signaling			
PI3K/AKT signaling	**1.7 × 10** ^**-5**^/ 3.6 × 10^-1^/ **6.9 × 10** ^**-4**^	49/ 15/ 34	0.0002
Insulin receptor signaling	**6.5 × 10** ^**-5**^/ 8.6 × 10^-1^/ **5.8 × 10** ^**-4**^	49/ 13/ 36	0.0002
Hypoxia signaling in the cardiovascular system	**6.5 × 10** ^**-5**^/ 4.5 × 10^-1^/ **6.9 × 10** ^**-4**^	30/ 9/ 21	0.0002
Chronic myeloid leukemia signaling	**1.5 × 10** ^**-4**^/ 4.5 × 10^-1^/ **1.7 × 10** ^**-3**^	38/ 12/ 26	0.0002
Production of NO and ROS in macrophages	**1.5 × 10** ^**-4**^/ N. A./ **4.3 × 10** ^**-6**^	62/ 9/ 53	0.0002
Pancreatic adenocarcinoma signaling	**1.5 × 10** ^**-4**^/ 7.6 × 10^-1^/ **7.4 × 10** ^**-4**^	42/ 12/ 30	0.0134
ILK signaling	**1.9 × 10** ^**-4**^/ 8.8 × 10^-1^/ **5.8 × 10** ^**-4**^	62/ 15/ 47	0.0002
AMPK signaling	**3.1 × 10** ^**-4**^/ 7.7 × 10^-1^/ **1.6 × 10** ^**-3**^	48/ 14/ 34	0.0026
JAK-STAT signaling	**3.1 × 10** ^**-4**^/ 8.8 × 10^-1^/ **6.9 × 10** ^**-4**^	31/ 7/ 24	0.0154
Renal cell carcinoma signaling	**3.1 × 10** ^**-4**^/ 6.8 × 10^-1^/ **2.1 × 10** ^**-3**^	30/ 9/ 21	0.0170
Ceramide signaling	**3.8 × 10** ^**-4**^/ 9.2 × 10^-1^/ **6.9 × 10** ^**-4**^	33/ 7/ 26	0.0026
mTOR signaling	**5.4 × 10** ^**-4**^/ 1.1 × 10^-2^/ 6.9 × 10^-2^	60/ 26/ 34	0.0059
CTLA4 signaling in cytotoxic T lymphocytes	**6.2 × 10** ^**-4**^/ 5.0 × 10^-2^/ 3.8 × 10^-2^	33/ 14/ 19	0.0002
B cell receptor signaling	**7.4 × 10** ^**-4**^/ 9.2 × 10^-1^/ **5.8 × 10** ^**-4**^	56/ 12/ 44	0.0002
Prostate cancer signaling	**7.9 × 10** ^**-4**^/ 9.2 × 10^-1^ **/ 8.5 × 10** ^**-4**^	32/ 7/ 25	0.0002
Glioma signaling	**7.9 × 10** ^**-4**^/ 4.5 × 10^-1^/ **7.6 × 10** ^**-3**^	36/ 12/ 24	0.0085
Non-small cell lung cancer signaling	**1.4 × 10** ^**-3**^/ 9.2 × 10^-1^/ **1.5 × 10** ^**-3**^	27/ 6/ 21	0.0002
Cyclins and cell cycle regulation	**2.0 × 10** ^**-3**^/ 1.9 × 10^-1^/ 4.1 × 10^-2^	30/ 12/ 18	0.0002
PDGF signaling	**2.0 × 10** ^**-3**^/ 9.0 × 10^-1^/ **1.7 × 10** ^**-3**^	30/ 7/ 23	**0.1620**
Reduced Protection against Oxidative Stress			
NRF2-mediated oxidative stress response	**3.5 × 10** ^**-5**^/ 8.7 × 10^-1^/ **8.5 × 10** ^**-4**^	60/ 17/ 43	0.0489

The pathway “mTOR Signaling” is assigned to two signatures: “Erythrocyte-Reticulocyte Ratio Shift” (positively associated transcripts) and “Attenuated Insulin Signaling” (negatively associated transcripts). Benjamini-Hochberg-FDR-values < 0.01 are provided in bold. Permutation analysis was performed to evaluate robustness of the pathway enrichment. The single Benjamini- Hochberg corrected *p*-value > 0.05 possibly indicating a false positive result (PDGF signaling) is underlined

### A gene expression signature indicating a ratio shift from mature erythrocytes towards reticulocytes

A large part of the 171 positively correlated gene-specific transcripts in the top 25 IPA-pathways specified components of the cellular protein synthesis apparatus (ribosomal proteins and translation factors) and mitochondrial proteins, in particular of the respiratory chain and the F_0_/F_1_ ATP synthase complex. This was mainly the case for the pathways *EIF2 signaling*, *Mitochondrial dysfunction*, *Oxidative phosphorylation*, *Regulation of eIF4 and p70S6K signaling*, and *mTOR signaling* (Fig. 2 and Table 2). Expression of these genes is strongly induced by pro-proliferative signals and mainly driven by the transcription factor MYC [13, 14]. The BMI-associated increase in the abundance of these transcripts most probably reflected a ratio shift from mature erythrocytes that contain only mRNA remnants [15] towards reticulocytes with substantial mRNA content. The number of bone marrow-residing adipocytes increases with BMI and adipocyte-secreted leptin promotes hematopoiesis and lymphopoiesis [16–18]. Thus, positive correlations were described between both red and white blood cell counts on the one hand and BMI/obesity as well as pre-diabetes/T2D [19, 20] on the other hand. In line with these findings, we observed significant positive correlations of serum leptin, fasting glucose, and 2 h-OGTT glucose with BMI in our study (*p* = 4.27 × 10^−245^, 1.23 × 10^−27^, and 3.25 × 10^−27^, respectively). Concurrently, erythrocyte survival decreases with increasing BMI and higher blood glucose concentration [21, 22], most probably due to pronounced oxidative stress [23]. Thus, the positive correlations between BMI and mRNA abundances of MYC-regulated genes of the *EIF2 signaling*, *Mitochondrial dysfunction*, *Oxidative phosphorylation*, *Regulation of eIF4 and p70S6K signaling*, and *mTOR signaling* are rather attributable to a shift in the erythrocyte/reticulocyte ratio than being a consequence of altered gene regulation, which might also be the case for many other positively correlated transcripts. This interpretation is strengthened by our observation that mRNA abundances of all seven genes of the *Heme biosynthesis II* pathway, which are highly expressed in reticulocytes, were also positively correlated with BMI (Additional file 2: Table S28).

### A gene expression signature indicating decreased expression of genes essentially involved in transmission and amplification of the insulin signal

**Fig. 3:**
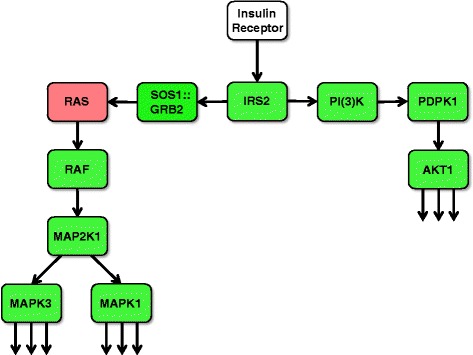
BMI-associated net down-regulation of the two main signal transduction axes propagating and amplifying the initial insulin signal. Ligand-activated insulin receptor mediates activating tyrosine phosphorylation of IRS (insulin receptor substrate) proteins. Downstream of IRS, there are two major signal transduction branches: The PI(3)K → PDPK1 → AKT (right) and the SOS1::GRB2 → RAS → RAF → AP2K1 (MEK family) → MAPK3/MAPK1 (ERK family) kinase cascades (left). Tyrosine-phosphorylated IRS proteins bind PI(3)K and the SOS1::GRB2 complex. The activated SOS1::GRB2 complex promotes GDP-GTP exchange on p21ras (RAS), thereby activating the RAS → RAF → MAP2K1 (MEK family) → MAPK3/MAPK1 (ERK family) branch. Activation of PI(3)K (phosphatidylinositol-4, 5-bisphosphate 3–kinase) by IRS causes the production of PI3,4P_2_ (phosphatidylinositol-3, 4-bisphosphate) and PI3,4,5P_3_ (phosphatidylinositol-3, 4,5-triphosphate) which recruit PDPK1 (3-phosphoinositide-dependent protein kinase 1) and AKT proteins to the membrane. Subsequently, AKT is activated by PDPK1-mediated tyrosine phosphorylation. Finally, AKT and the ERK family kinases phosphorylate numerous cellular substrate proteins, resulting in their activation or inactivation (for details see Additional file 1: Supplementary Materials S1). Green and red coloring indicates signal transduction modules with corresponding mRNA levels that are negatively and positively correlated, respectively, with BMI in the present study

**Fig. 4:**
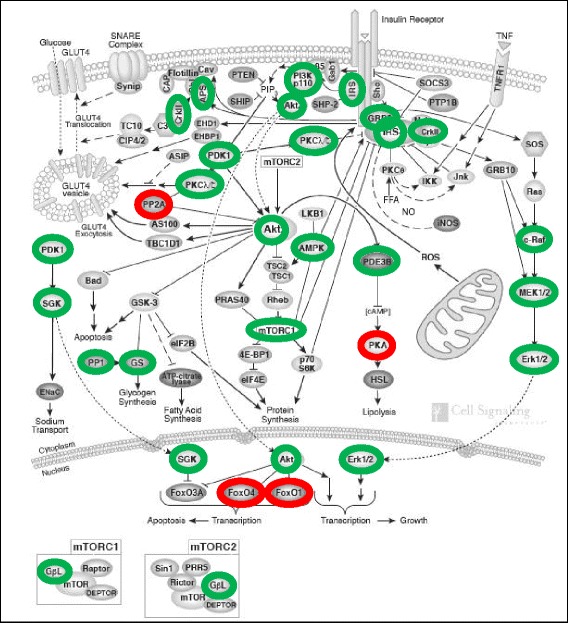
Attenuation of insulin signaling under conditions of increased BMI as derived from the mRNA abundances as determined in the present study. Green and red coloring indicates negative and positive correlation of the gene-specific transcripts specifying the corresponding proteins/protein complexes shown here with BMI. Labeled are those insulin signaling modules whose decrease or increase on the protein level is predicted to contribute to or to be indicative of attenuated transduction of the insulin signal. Illustration adopted and modified by courtesy of Cell Signaling Technology/New England Biolabs

The majority of gene-specific transcripts of the other 20 pathways which share a large number of genes (Fig. 1) were negatively correlated with BMI. Strikingly, five genes (*IRS2*, *PIK3CD*, *PIK3R4*, *PDPK1*, *AKT1*) encoding key proteins involved in AKT-dependent transduction of the insulin signal exhibited decreased mRNA abundances with increasing BMI (Fig. 3; for details see Additional file 1: Supplementary Materials S1). Besides the AKT-linked signal transduction cascade, the insulin signal is also transmitted by the SOS1::GRB2-RAS-RAF-MEK axis. This pathway primarily mediates the initiation of mRNA translation as well as of transcriptional responses (Fig. 3; for details see see Additional file 1: Supplementary Materials S1). Five genes (*GRB2*, *RAF1*, *MAP2K1*, *MAPK3*, *MAPK1*) encoding key proteins of this latter signaling axis showed decreased mRNA levels with increasing BMI. Regulation of the genes of these two main insulin signaling pathways is particularly important as strong amplification of the primary insulin signal occurs during these initial transmission steps. Furthermore, 26 additional genes involved in insulin signaling downstream of the main axes exhibited reduced mRNA abundances with increasing BMI. These expression changes could also contribute to or at least indicate attenuated signaling. In contrast, BMI-transcript correlations indicating improved signaling could only be detected for five additional genes (for details see Fig. 4 and Additional file 1: Supplementary Materials S1).

We further observed positive correlations between *FOXO3* or *FOXO4* mRNA abundances and BMI. Both genes encode prominent transcription factors involved in glucose homoeostasis. As a consequence of decreased AKT-dependent phosphorylation, increased amounts of FOXO proteins translocate to the nucleus where they drive the expression of their own genes in a positive feedback loop. This mechanism may explain the positive correlation of *FOXO3* or *FOXO4* transcript levels with BMI.

The mRNA signature of attenuated insulin signaling suggests that high BMI may cause lower amounts of key proteins involved in transduction and amplification of the insulin signal, pointing to a plausible mechanism contributing to the well-established association between obesity and insulin resistance. In line with these findings, we also found positive correlations between BMI and fasting glucose (*p* = 1.23 × 10^−27^), 2 h-OGTT glucose (*p* = 3.25 × 10^−27^), blood insulin (*p* = 1.11 × 10^−14^) as well as HOMA-IR (*p* = 3.95 × 10^−18^), confirming the link between increased BMI/obesity and insulin resistance or (pre)diabetes, respectively. The demonstration of attenuated insulin signaling processes in easily accessible whole-blood samples was surprising as blood cells do not belong to the classical insulin-sensitive tissues primarily involved in glucose homoeostasis, such as liver, skeletal muscle, and adipose tissue.

### A gene expression signature indicating reduced expression of key genes involved in the defence against reactive oxygen species (ROS)

**Fig. 5:**
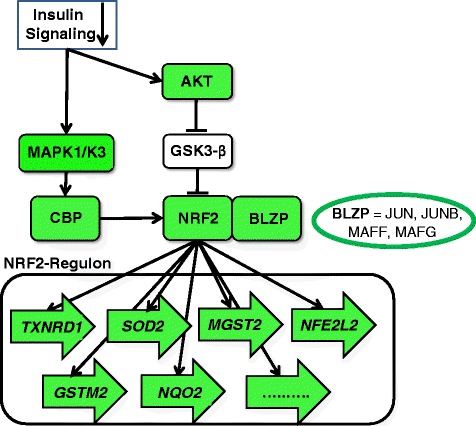
BMI-associated down-regulation of the NRF2 regulon. The transcription factor NRF2, after translocation from the cytoplasm to the nucleus, binds to the *cis*-acting enhancer sequence ARE (Antioxidant Response Element) located in promoters upstream of several genes encoding proteins necessary for glutathione synthesis and electrophile detoxification (for details see Additional file 1: Supplementary Materials S3). Transcriptional activation of ARE-mediated genes requires hetero-dimerization of NRF2 with other basic leucine zipper proteins (BLZP), namely JUN (c-JUN, JUN-D and JUN-B) and the small musculoaponeurotic fibrosarcoma (MAF) proteins (MAFG, MAFK and MAFF). In addition, the *CREBBP* encoded CBP protein [cAMP-response element binding protein (CREB) binding protein], a histone acetyl transferase, represents a nuclear co-activator directly trans-activating NRF2. Among the prominent ARE-mediated NRF2 targets genes are *GSTM2* and *MGST2* encoding the glutathione S-transferases μ2 and microsomal glutathione S-transferase 2, respectively, the *NQO2*-encoded NAD(P)H:quinone acceptor oxidoreductase 2, the *SOD2*-encoded manganese-containing superoxide dismutase 2 (MnSOD), and the *TXNRD1*-encoded thioredoxin reductase 1. In addition, the NRF2 encoding gene *NFE2L2* contains two ARE-like sequences in its promoter and is therefore auto-regulated by NRF2 in a positive feedback-loop. Green coloring indicates that the corresponding mRNA abundances were negatively correlated with BMI in the present study. Arrows indicate ARE-regulated genes

The pathomechanisms underlying vascular and non-vascular complications related to insulin resistance, metabolic syndrome and T2D have not been completely resolved yet. Recent epidemiological evidence suggests a central role of oxidative stress [24, 25]. In our analyses, the transcript levels of multiple prominent target genes of NRF2, the key regulatory transcription factor of the major cellular defense system against oxidative stress, were negatively correlated with BMI (Fig. 5; for details see Additional file 1: Supplementary Materials S3). Consistent with this finding, mRNA levels of four genes encoding direct NRF2 dimerization partners and of *CREBBP* encoding a nuclear trans-activator of NRF2 were also negatively correlated with BMI (Fig. 5). Furthermore, full activation of NRF2 requires insulin signaling *via* the AKT- and ERK-mediated phosphorylation cascades, which are predicted to be attenuated with increasing BMI. Together these results indicate that the most important cellular defense system against oxidative stress becomes progressively impaired with increasing BMI, which is in agreement with the aforementioned epidemiological associations.

## Conclusions

Here, we demonstrate extensive BMI-associated alterations of the whole-blood transcriptome with more than 3,500 transcripts exhibiting positive or negative BMI-correlation. Of particular interest, we report the identification of three distinct gene expression signatures associated with increased BMI in a meta-analysis of array-based whole-blood transcriptome profiling data of 1977 individuals from two large population-based cohorts. These signatures reflect (1) a ratio shift from mature erythrocytes towards reticulocytes, (2) impaired insulin signaling, and (3) impaired defense against oxidative stress. The latter two signatures corroborate and augment epidemiological findings and emphasize the value of whole-blood gene expression profiling for the analysis of molecular mechanisms underlying complex traits and diseases. Comparing the correlations between detected transcripts and BMI with the correlations between transcripts and five further phenotypes of relevance in the context BMI/ insulin resistance revealed pronounced overlaps in the order of decreasing similarity: BMI > serum leptin > 2 h-OGTT glucose > fasting glucose > blood insulin > HOMA-IR. This was consistent for all three expression signatures found in our analyses (Additional file 5: Figure S1). As circadian rhythms in whole-blood gene expression patterns have been described [26], it has to be emphasized that in SHIP-TREND as well in KORA F4 all blood samples were collected between 8 a.m. and noon from fasting individuals. Considerable bias of our results due to circadian blood cell gene expression variation is thus unlikely.

The mechanisms underlying the extensive BMI-associated down-regulation of several key genes involved in insulin signaling and defense against oxidative stress on the mRNA level are currently unclear. Interestingly, several micro-RNAs (miRNAs) have been found to be differentially expressed in tissues relevant for insulin signaling and resistance (liver, skeletal muscle, adipose tissue, and pancreatic beta cells) during obesity, hyperglycemia, and diabetes (for details see see Additional file 1: Supplementary Materials S4). It is conceivable that the BMI-associated down-regulation of many genes may be mediated by specific miRNAs.

One limitation of this study is the fact that it is exclusively based on gene expression profiles obtained from whole-blood cell analyses in a cross-sectional design. The data indicating attenuated insulin signaling and decreased resistance against oxidative stress might not directly translate to other tissues more relevant for insulin signaling such as liver, skeletal muscle and adipose tissue. Although our results already have gained some support by observations in animal models [27, 28], appropriate functional analyses are needed to reveal the details of the molecular mechanisms involved. A further limitation consists in the fact that the observed BMI-associated mRNA level alterations represent two co-occurring phenomena, specific gene expression changes on the one hand but also the described erythrocyte-reticulocyte shift on the other hand. Due to the fact that reticulocyte counts were determined neither in SHIP nor in KORA, an appropriate adjustment was not possible. Therefore, the interference of both BMI-dependent effects, namely the decreased amounts of specific mRNAs due to reduced gene expression and the increased amounts of other mRNAs due to a higher reticulocyte number, may explain seemingly contradictory observations, which can be illustrated using NRF2-regulated genes as an example. Although nearly all genes encoding NRF2 key regulators (including the structural gene of the transcription factor itself, *NFE2L2*) as well as some of the most prominent NRF2 targets exhibited a negative mRNA correlation with BMI, some other target gene transcripts were positively correlated. These latter genes might be particularly strongly expressed in reticulocytes, overriding their down-regulation in leukocytes. A generalization of this assumption would imply that the effect strengths of other observed negative correlations between BMI and transcripts, as those observed for the genes encoding insulin signaling key proteins, might be even more pronounced in isolated leukocytes, in particular neutrophil granulocytes, as these represent the predominant white cell fraction in whole-blood. Therefore, while a transcriptome analysis of isolated granulocyte fractions would be most desirable in future studies addressing associations between BMI and gene expression, whole-blood expression patterns are also of value but likely underestimate the true expression changes occurring in different white blood cell populations, including granulocytes. It also has to be mentioned that with a mean age of 50 and 70 years in KORA and SHIP, respectively, these cohorts are relatively old and thus, we cannot exclude the possibility that at least partially differing results would be obtained with clearly younger individuals. As the relationship between obesity and mortality changes with increasing age, the identified gene expression signatures, in particular those related to insulin signaling and oxidative stress defense, might also be affected in a younger cohort. Finally, we would like to emphasize the fact that the three extracted signatures are based on a subset of around 400 from the more than 3700 BMI-associated gene-specific transcripts annotated in IPA. This demonstrates that for most of the BMI-associated whole-blood transcriptome, adequate physiological interpretation is still not available. Further hypotheses about the mechanisms underlying the observed associations might be generated by using more sophisticated bioinformatical approaches in future analyses.

### Availability of supporting data

The KORA F4 whole-blood transcriptome raw data are deposited in the ArrayExpress Archive of Functional Genomics Data (https://www.ebi.ac.uk/arrayexpress/) with the accession number E-MTAB-1708. The SHIP-TREND whole-blood transcriptome raw data are deposited in the GEO (Gene Expression Omnibus) public functional genomics data repository (http://www.ncbi.nlm.nih.gov/geo/) with the GEO series accession number GSE36382.

## Additional files

Additional file 1: **Supplementary Materials: Supplementary Text: Supplementary information about relevant pathways and genes.** Supplementary References. Legend for supplementary Figure. (DOC 315 kb)

Additional file 2: Table S1. Results of the SHIP-TREND/KORA F4 meta-analysis with p (BH) < 0.01 (z-score based p-value). **Table S2.** Number of probes indicating significantly BMI-associated expression in the SHIP-TREND/KORA F4 meta-analysis. **Table S3.** Number of genes exhibiting significantly BMI-associated expression in the SHIP-TREND/KORA F4 meta-analysis. **Table S4.** Genes exhibiting BMI-associated mRNA abundances and are assigned to the 25 most significantly BMI-associated canonical IPA-pathways detected in this study. **Table S5.** BMI-associated pathways with p (BH) < 0.05. **Tables S6-S30.** Genes exhibiting BMI-associated mRNA abundances and are assigned to the canonical IPA-pathways listed in Table 2. **Table S31.** Annotation of all 48,803 Illumina HumanHT-12 v3 probes and respective detection rates (Illumina’s detection p-value < 0.05) in SHIP-TREND and KORA F4. **Table S32.** Meta-analysis results for the main model with and without adjustment for HOMA-IR for all 12,778 Illumina probes indicating significant transcript levels in SHIP-TREND and KORA F4. **Table S33.** Association results for the detected genes priorized using the DEPICT method and described in Locke et al. [5]. **Table S34.** Results of sensitivity analyses in KORA F4 - additional adjustment for HOMA-IR or type 2 diabetes status. (XLSX 18052 kb)

Additional file 3: **Supplementary Table KEGG_pathways_WebGestalt: Results of the pathway over-representation analysis using the web-based toolkit WebGestalt (** **bioinfo.vanderbilt.edu/webgestalt/** **) and the online database resource KEGG (** **genome.jp/kegg/** **).** (XLS 46 kb)

Additional file 4: **Supplementary Table WikiPathways_pathways_WebGestalt: Results of the pathway over-representation analysis using the web-based toolkit WebGestalt (** **bioinfo.vanderbilt.edu/webgestalt/** **) and the online database resource** ***WikiPathways*** **(** **wikipathways.org** **).** (XLS 39 kb)

Additional file 5: Figure S1. Correlation of Signature Transcripts with BMI-related Traits. (PDF 39 kb)

## Abbreviations

BMI: Body mass index
FDR: Benjamini and Hochberg false discovery rate
GWAS: Genome-wide association study
HOMA-IR: Homeostasis model assessment – insulin resistance
KORA: Kooperative Gesundheitsforschung in der Region Augsburg (Cooperative Health Research in the Region of Augsburg)
RBC: Red blood cell count
RIN: RNA integrity number
ROS: Reactive oxygen species
SHIP: Study of Health in Pomerania
T2D: Type 2 diabetes mellitus
TWAS: Transcriptome-wide association study
WBC: White blood cell count
