# Supplementary material for: Extensive alterations of the whole-blood transcriptome are associated with body mass index: results of an mRNA profiling study involving two large population-based cohorts
Source: BMC Med Genomics. 2015 Oct 15;8:65. doi: 10.1186/s12920-015-0141-x (PMC4608219; doi:10.1186/s12920-015-0141-x)
Supplement: Supplementary file 1 — Supplementary Materials: Supplementary Text: Supplementary information about relevant pathways and genes. Supplementary References. Legend for supplementary Figure. (DOC 315 kb) [file 12920_2015_141_MOESM1_ESM.doc]

**Extensive Alterations of the Whole-Blood Transcriptome are associated with Body Mass Index: Results of an mRNA Profiling Study involving two large Population-based Cohorts**

**SUPPLEMENTARY MATERIALS**

**SUPPLEMENTARY METHODS: EVALUATING ROBUSTNESS OF INGENUITY PATHWAY ANALYSIS BY PERMUTATION STATISTICS**

**SUPPLEMENTARY TEXT: SUPPLEMENTARY INFORMATION ABOUT RELEVANT PATHWAYS AND GENES.**

S1. Genes and proteins involved in insulin signaling: The two main axes of insulin signaling (AKT-dependent and RAS-RAF-dependent).

S2. Additional genes and proteins involved in insulin signaling and glucose homeostasis.

S3. Genes belonging to the major cellular defense system against oxidative stress, the NRF2 regulon.

S4. Selected literature concerning micro-RNAs (miRNAs) differentially expressed in insulin signaling- and insulin resistance-relevant tissue (liver, skeletal muscle, adipose tissue, and pancreatic beta cells) during obesity, hyperglycemia, and diabetes.

**SUPPLEMENTARY REFERENCES**

**LEGEND FOR SUPPLEMENTARY FIGURE**

**SUPPLEMENTARY METHODS: EVALUATING ROBUSTNESS OF INGENUITY PATHWAY ANALYSIS BY PERMUTATION STATISTICS**

Permutation analysis was performed for evaluating robustness of Ingenuity Pathway Analysis (IPA) and identifying potentially false positive over-representations. All gene-pathway mappings from the IPA online database were downloaded. According to the number of significantly BMI associated genes, 3,762 genes were randomly selected 5,000 times from the list of all genes expressed in whole-blood and mapped to IPA pathways. Only pathways with more than two BMI associated genes were considered in further analysis. For each pathway, over-representation Z scores, a permutation p-value and Benjamini-Hochberg corrected p-values were calculated as described by Zambon *et al*. .

**SUPPLEMENTARY INFORMATION ABOUT RELEVANT PATHWAYS AND GENES.**

*1. Genes and proteins involved in insulin signaling: The two main axes of insulin signaling (AKT-dependent and RAS-RAF-dependent)*

***IRS2*** encodes the **insulin receptor substrate 2** which is the first protein that interacts with the ligand-activated, auto-phosphorylated insulin receptor (IR) and is subsequently tyrosine-phosphorylated and activated by the former.

***PIK3CD***, ***PIK3R2***, and ***PIK3R4*** encode different subunits of the **phosphatidylinositol-4,5-bisphosphate 3–kinase (PI(3)K)**: the catalytic Δ-subunit (p110) primarily expressed in leukocytes and the regulatory subunits 2 (β, p85B) and 4 (p150). The corresponding functional PI(3)K proteins, either the p110::p85B or the p110::p150 dimer, activated by interaction of two SH2 domains within their regulatory subunits and tyrosine-phosphorylated motifs in the activated IRS proteins, subsequently catalyze the phosphorylation of membrane phosphoinositides on their 3-position, resulting in increased amounts of phosphatidylinositol-3-phosphates, especially phosphatidylinositol-3,4,5-triphosphate (PtdIns(3,4,5)P_3_).

***PDPK1*** encodes the **3-phosphoinositide-dependent protein kinase 1**. *Via* its pleckstrin homology (PH) domain, this kinase is tethered to the PtdIns(3,4,5)P3 molecules that are present in the membrane in increased amounts under conditions of stimulated insulin signaling.

This also holds true for the **AKT kinase** encoded by ***AKT1*** which possesses a PH domain too. After insulin stimulation, PDPK1 and AKT are trans-located in large amounts to the membrane *via* PtdIns(3,4,5)P_3_ binding. This close co-localization allows for activation of AKT by PDPK1-mediated tyrosine phosphorylation .

Activated AKT represents a key module in the transmission of the insulin signal as it phosphorylates many cellular substrates fulfilling essential functions in insulin signaling, among these GSK3α/β, AS160, the BAD::BCL2 heterodimer, the FOXO transcription factors, p21^CIP1^ and p27^KIP1^, eNOS, PDE3B, and TSC2. This results in blocking of glycogen synthesis inhibition (and consequently induction of glycogenesis) as well as in the stimulation of protein biosynthesis, promotion of translocation of the GLUT4 glucose transporter from intracellular sites to the membrane (and consequently stimulation of glucose uptake), inhibition of apoptosis, inhibition of tissue-specific gene expression (e. g. of liver-specific genes encoding enzymes involved in gluconeogenesis), blocking of cell cycle inhibition, stimulation of NO synthesis (and consequently of vasodilatation), stimulation of cyclic AMP (cAMP) hydrolysis (and, *via* inactivation of cAMP response element binding (CREB) protein, inhibition of gluconeogenesis and fatty acid oxidation), and activation of the mTOR1 pathway (and consequently in stimulation of lipogenesis and protein synthesis), respectively .

The **R-RAS protein** represents a small G protein of the Ras family attached to the membrane by a lipid anchor. R-RAS is activated by the **SOS1::GRB2 protein complex**. **GRB2**, *via* its SH2 domain, interacts with the tyrosine-phosphorylated, activated IRS protein and is thereby activated itself. Subsequently, it stimulates the nucleotide exchange activity of SOS1. This causes the replacement of R-RAS-bound GDP by GTP, thereby activating R-RAS.

Activated R-RAS stimulates the **RAF1 MAP kinase kinase kinase (MAP3K)** encoded by ***RAF1***. Subsequently, activated RAF1 phosphorylates the **MEK kinases 1 (MAP2K1)** and 2 (MAP2K2), thereby activating both.

The **MEK-1** protein encoded by ***MAP2K1*** and the MEK-2 protein phosphorylate and activate the members of the **ERK (extracellular-signal-regulated kinases) family**, defined as **ERK1 (MAPK3)** **and ERK2 (MAPK1)**. ERK1 and ERK2 are encoded by ***MAPK3*** and ***MAPK1***, respectively. Activated ERKs also represent important modules in the transmission of the insulin signal as they phosphorylate many cellular substrates with crucial functions in insulin signaling, including the transcription factors ELK1, MYC, UBF, and FOS as well as the MAP kinase interacting serine/threonine kinases 1 and 2 (MKNK1 and MKNK2), resulting in the transcriptional induction of many downstream genes (among them *FOS* itself and those genes encoding ribosomal proteins) as well as in initiation of translation by activating phosphorylation of eukaryotic translation initiation factor 4E (EIF4E) .

*2. Additional genes and proteins involved in insulin signaling and glucose homeostasis*

The **ATM kinase** whose activity significantly increases after insulin stimulation phosphorylates 4EBP1 (also called PHAS-I), an insulin-responsive cytoplasmic protein, which results in dissociation of this protein from eIF4E (eukaryotic translation initiation factor 4E). As eIF4E represents a crucial component of the multi-subunit complex that recruits 40S ribosomal subunits to the 5'-ends of mRNAs, inhibition of eIF4E by association with 4EBP1 results in a global repression of translation. Therefore, ATM-dependent inactivating phosphorylation of 4EBP1 strongly enhances the cellular protein synthesis capacity . Furthermore, ATM stimulates insulin-induced AKT phosphorylation and therefore mediates the full activation of AKT activity. Decreased amounts of ***ATM***-specific mRNA that were observed under conditions of increased BMI can therefore be predicted to contribute to the down-regulation of insulin signaling in general and to an attenuation of insulin-stimulated protein synthesis in particular.

Binding of the lipid messenger phosphatidic acid (PA) stimulates the mTOR kinase complex and, independently of mTOR, the S6K kinase (see below) . By phosphorylation of diacylglycerol, diacylglycerol kinase zeta (DGKζ) encoded by *DGKZ* produces PA, resulting in positive regulation of mTOR activity . Hydrolysis of the terminal diester bond of glycerophospholipids also causes formation of PA. **Phospholipase D2** encoded by ***PLD2*** catalyzes this reaction, thus also stimulating mTOR activity . Therefore, the lower amounts of *PLD2* specific mRNA under conditions of increased BMI might contribute to attenuated mTOR-mediated insulin-stimulated protein synthesis.

The gene product of ***CRK*** represents an **adaptor protein**. *Via* its SH2 domain it binds to the activated, mono-phosphorylated PDPK1 attached to PtdIns(3,4,5)P_3_ molecules that are present in the membrane in higher amounts after insulin stimulation. Subsequently, CRK mediates full phosphorylation of PDPK1 by IRS proteins, resulting in its maximal PDPK1 activation . Therefore, the low amounts of ***CRK***-specific mRNA correlated with increased BMI should cause decreased PDPK1 activity, resulting in attenuated downstream insulin signaling.

Similarly, the protein encoded by ***CRKL*** represents a **typical adaptor protein** that links, *via* its SH2- and SH3-domains, activated IRS receptors with downstream signaling modules . Lower amounts of *CRKL* mRNA under conditions of increased BMI may therefore contribute to attenuated insulin signaling.

**Protein phosphatase 2 (PP2A)** consists of a dimeric core composed of structural A and catalytic C subunits, as well as a regulatory B subunit. The members of the regulatory B subunit protein family, exhibiting pronounced sequence heterogeneity, are specifying localization and activity of the different holoenzymes . Of the genes *PPP2CA* and *PPP2CB* encoding catalytic subunits, the former exhibited a negative correlation of its corresponding mRNA amounts with BMI, while the latter did not show BMI-dependency. Of the two genes that encode structural subunits, *PPP2R1A* exhibited a negative correlation with BMI, whereas the amount of *PPP2R1B* mRNA was not correlated with BMI. Of the genes encoding regulatory subunits, *PPP2R2A*, *PPP2R3B*, and *PPP2R5D* showed a negative correlation with BMI, whereas correlation was positive for *PPP2R2B* and ***PPP2R5B***. The latter finding was of particular interest in the given context: CLK2 kinase-dependent phosphorylation of the **PP2A regulatory subunit B56β (or B’β)** encoded by *PPP2R5B* represents a critical regulatory step in the assembly of the PP2A holoenzyme complex on AKT, resulting in inactivating AKT dephosphorylation and consequently in the attenuation of the insulin signal . Therefore, the observed increase in the amount of *PPP2R5B* mRNA might, *via* increased levels of the encoded subunit, contribute to further down-regulation of the already alleviated insulin signaling.

The protein encoded by ***IRS4*** shares the common overall architecture of the members of the IRS family. It can be phosphorylated after insulin- and IGF-1 stimulation, binds to SH2 domain-containing proteins including PI(3)K and Grb-2, and promotes several biological actions of insulin and IGF-1 . However, mice lacking IRS4 do not show an apparent phenotype, and it was suggested that IRS4 (and IRS3) may act as a negative regulator of the IGF-1/Insulin signaling pathway by suppressing the function of other IRS proteins, namely IRS1 and IRS2 . The increased amounts of *IRS4* specific mRNA under conditions of high BMI could therefore contribute to the general down-regulation of insulin signaling.

***PRKCD*** encodes the **novel** **protein kinase C (PKC)-δ** belonging to a family of serine–threonine kinases (PKC) that play important regulatory roles in a variety of biological phenomena. In skeletal muscle, insulin induces tyrosine phosphorylation, translocation, and activation of PKCs α, βII, δ, and ζ. Whereas activation of PKCs βII and ζ occurs *via* a PI3-kinase- and PDPK1-dependent pathway, PKCs δ and α are activated within 1–5 min independently of PI3 kinase. This activation involves participation of the tyrosine kinase Src. As demonstrated in hepatocytes, PKC-δ is constitutively physically associated with the IR, where this association is increased by insulin stimulation and regulates subsequent events in IR signaling such as IR tyrosine phosphorylation and IR internalization. Furthermore, insulin-stimulated PKC-δ plays an essential role in AKT-activation and inhibitory GSK3 phosphorylation as well as in insulin-induced glucose uptake and glycogen synthesis . Consequently, the decreased amounts of *PRKCD* mRNA at increased BMI can be predicted to contribute to the globally attenuated insulin signaling under these conditions as well as, more specifically, to the well-known associated insulin resistance phenotype.

***PRKCZ*** encodes the **atypical protein kinase C (PKC)-ζ** which is activated by insulin *via* PDPK1-dependent phosphorylation and subsequently mediates GLUT4-mediated glucose uptake . Furthermore, PKC-ζ activates the members of the so-called p70 ribosomal S6 kinase (p70S6K) family, namely S6K1 and S6K2 encoded by *RPS6KB1* and *RPS6KB2*, respectively. Activated p70S6K phosphorylate the ribosomal protein S6, thereby facilitating mRNA translation, especially of mRNA molecules exhibiting large stretches of pyrimidine residues within their 5’-UTRs. Such stretches are typical characteristics of mRNAs specifying ribosomal proteins. Consequently, PKC-ζ activation increases the cellular protein synthesis capacity . The decreased amounts of *PRKCZ*-specific mRNA that we observed under conditions of increased BMI could therefore contribute to high-BMI-induced insulin resistance as well as to the attenuation of insulin-stimulated protein synthesis. In this context, the increased amounts of *RPS6KB2* mRNA correlated with high BMI might represent a compensatory transcriptional up-regulation, as it should *per se* cause improved insulin signaling.

The **SH2B2 adaptor protein**, formerly known as **APS**, represents a further substrate of the IRS proteins. The interaction with insulin-activated IRS is mediated by the SH2B2 SH2 domain, which is tyrosine-phosphorylated by IRS and subsequently serves as a binding site for the SH2 domain of the CBL protein. Thus, activating phosphorylation of CBL by IRS is strongly facilitated, resulting in activation of a pathway that includes several further proteins (TC10, CBL-associated protein, CRK, C3G) and finally mediates plasma membrane fusion of GLUT4-containing vesicles, thereby facilitating glucose uptake as described for skeletal muscle . Consequently, the decreased amounts of ***SH2B2*** mRNA at increased BMI could be predicted to contribute to the well-known insulin resistance phenotype observed under these conditions.

Insulin-stimulated PDK1-dependent phosphorylation activates **serum/glucocorticoid regulated kinase 1 (SGK1)** encoded by ***SGK1****.* Active SGK1 phosphorylates many target proteins, including transporters like the glucose transport proteins SGLT1, GLUT1, and GLUT4 as well as several amino acid transporters, thereby promoting glucose and amino acid uptake in different tissues . The decreased *SGK1* mRNA levels correlated with increased BMI might therefore contribute to glucose intolerance due to alleviated transporter activation.

Upon insulin-stimulation, **protein phosphatase 1 (PP1)** consisting of one catalytic and one regulatory subunit dephosphorylates many target proteins, resulting in their activation or inactivation. Importantly, PP1 mediates activating glycogen synthase (GS) dephosphorylation and inactivating glycogen phosphorylase (PYG) dephosphorylation, resulting in the induction of glycogenesis . ***PPP1CA***, ***PPP1R3D***, ***PPP1R10***, ***PPP1R11***, ***PPP1R14B***, and ***PPP1R15A*** encode one catalytic and five regulatory subunits of PP1, respectively. All six genes exhibited decreased mRNA amounts under conditions of increased BMI, which, *via* decreased amounts of PP1, may attenuate insulin-induced glycogenesis.

In line with this finding, the coding gene of **skeletal muscle GS** itself, ***GYS1***, also exhibited lower mRNA amounts at higher BMI.

Insulin-stimulated, AKT-mediated activating phosphorylation of **phosphodiesterase 3B (PDE3B)** causes stimulation of cAMP hydrolysis and, *via* inactivation of CREB due to decreased cAMP levels, subsequent inhibition of hepatic gluconeogenesis: cAMP-activated CREB induces the gluconeogenic gene expression program through the nuclear receptor co-activator PGC-1α . This is mediated by cAMP-dependent protein kinase A (PKA) which is directly activated by cAMP binding and subsequently activates CREB by phosphorylation . In adipocytes, PDE3B-mediated cAMP reduction stimulates GLUT-4 translocation to the plasma membrane and therefore glucose uptake, as well as lipogenesis, whereas lipolysis is attenuated . In the present study, the ***PDE3B***-specific mRNA exhibited a negative correlation with BMI. Thus, increased cAMP levels due to lower amounts of PDE under these conditions can be derived, in turn causing higher activities of CREB and possibly PGC-1α. This notion is in line with the known finding of increasingly unrestricted hepatic gluconeogenesis under conditions of obesity-induced metabolic syndrome, pre-diabetes, and finally manifest diabetes, as well as impaired lipolysis and pronounced lipogenesis due to insulin resistance. Furthermore, constitutive activation of CREB due to increased cAMP levels in adipose tissue under obese conditions with promotion of insulin resistance by triggered expression of the transcriptional repressor ATF3 and subsequent down-regulated expression of the adipokine hormone adiponectin as well as of GLUT4 has been described .

A further finding can be hypothesized to strengthen these effects: the mRNA amounts specified by ***PRKAR1A*** encoding an **inhibitory regulatory subunit of PKA**  were also negatively correlated with BMI. The PKA holo-enzyme represents a tetramer composed of two catalytic and two regulatory subunits, where the latter form a dimer and determine the isotype of human PKA (type I and type II, respectively). Binding of cAMP to the regulatory subunits causes dissociation of the two catalytic subunits, resulting in their activation . Of note, among the four genes encoding regulatory subunits (*PRKAR1A*, *PRKAR1B*, *PRKAR2A*, and *PRKAR2B*), *PRKAR1A* specifies the highest amounts of mRNA in white blood cell preparations according to the *Illumina Body Map*, and this pattern was essentially reflected in skeletal muscle, liver and adipose tissue, too. Therefore, a lower amount of *PRKAR1A* specific mRNA under conditions of increased BMI can be predicted to result in higher PKA activity due to attenuated inhibition of the catalytic sub-units, and consequently in more pronounced activating phosphorylation of CREB.

The ***DDIT4*** (DNA damage-inducible transcript 4)-encoded **REDD1** protein (**regulated in development and DNA damage responses**) negatively regulates the activity of the mammalian target of rapamycin (mTOR) multiprotein complexes . The mTOR complexes one and two (mTOR1 and mTOR2) consist of several partially different sub-units: mTOR1 is composed of mTOR, mLST8, Raptor, and PRAS40, whereas mTOR2 is composed of mTOR, mLST8, Rictor, mSin1, and Protor . The mTOR complexes integrate several extrinsic signals regulating cell growth and metabolism. Among the major downstream targets of mTOR1 are the members of the so-called p70 ribosomal S6 kinase family, S6K1 and S6K2, which are encoded by *RPS6KB1* and *RPS6KB2*, respectively, as well as 4EBP1 (eukaryotic translation initiation factor 4E binding protein 1) which is encoded by *EIF4EBP1*. The activated p70 ribosomal S6 kinases phosphorylate the ribosomal protein S6, thereby facilitating mRNA translation especially of mRNA molecules exhibiting large stretches of pyrimidine residues within their 5’-UTRs. Such stretches are typical characteristics of mRNAs specifying ribosomal proteins . Furthermore, mTOR1-dependent phosphorylation of 4EBP1 causes dissociation of this protein from eIF4E (eukaryotic translation initiation factor 4E). As eIF4E represents a crucial component of the multi-subunit complex that recruits 40S ribosomal subunits to the 5'-ends of mRNAs, inhibition of eIF4E by association with 4EBP1 results in a global repression of translation. Therefore, mTOR1 signaling *via* phosphorylation of ribosomal protein S6 and 4EBP1 strongly enhances the cellular protein synthesis capacity . Both mTOR complexes are regulated by TSC1/TCS2, a GTPase-activating protein for the Ras-related small G protein RheB, which regulates mTOR activation (see below).

On the one hand, insulin represents a strong mTOR activator by mediating AKT-dependent inhibitory TSC2 phosphorylation. On the other hand, sustained mTOR activation contributes to the development of insulin resistance, where one of the mechanisms involved is the decrease of IRS tyrosine phosphorylation, concomitant with an increase in its serine phosphorylation. The mTOR complexes are among the kinases known to phosphorylate IRS directly on serine residues. REDD1 which represents one of several proteins involved in mTOR1 regulation is required for down-regulation of mTOR1 in response to hypoxia and sustained insulin signaling. REDD1 controls mTOR1 activity through 14-3-3 proteins which directly associate with TSC2, thereby inhibiting TSC2 activity. By binding to 14-3-3 proteins, hypoxia- or insulin-induced REDD1 relieves the TSC2 inhibition, resulting in mTOR1 inactivation. Transcriptional up-regulation of *DDIT4* in response to hypoxia and insulin is mediated by hypoxia-inducible factor 1α (HIF-1α) in a PI3K- and mTOR-dependent fashion, and it was hypothesized that mTOR directly phosphorylates HIF-1α or one of its upstream regulators, resulting in an increased HIF-1α stability and abundance .

In various cell types, insulin shares with hypoxia the ability to induce the transcription of genes associated with increased energy flux *via* HIF-1α/ARNT, a common basic helix–loop–helix-PAS transcription complex consisting of HIF-1α and the aryl hydrocarbon nuclear translocator (ARNT) . Increased *DDIT4* expression in response to insulin represents a regulatory loop to restore a basal signaling pathway, as prolonged insulin stimulation down-regulates insulin signaling by promoting inhibitory mTOR-dependent serine phosphorylation of IRS. Therefore, long-term induction of *DDIT4* expression by insulin represents a mechanism necessary to terminate the signal on mTOR, thereby restoring the stimulatory potential of insulin . The negative correlation of *DDIT4* mRNA with BMI might therefore indicate decreased HIF-1α-mediated expression of the gene due to attenuated AKT-dependent insulin signaling. In addition, the coding gene of **HIF-1α**, ***HIF1A***, also exhibited lower mRNA amounts at higher BMI, which should cause generally reduced HIF-1α-mediated insulin signaling.

The negative correlation of ***MLST8*** mRNA amounts with BMI can be predicted to result in generally decreased mTOR-mediated insulin signaling, as the encoded **mLST8** (**mTOR-associated LST8 homolog protein**) represents a constitutive subunit of both mTOR1 and mTOR2 (see above).

***ULK1*** encoding **unc-51-like kinase 1**, a master regulator of autophagy, also exhibited lower transcript amounts at increased BMI. Transcription of this gene is directly positively regulated by ATF4 . Autophagy as a highly regulated cellular degradation pathway represents an important cellular response to starvation and has been implicated in many diseases, including diabetes . During autophagy, a double membrane first surrounds cytoplasmic contents and organelles, such as mitochondria, endoplasmic reticulum or peroxisomes, resulting in the autophagosome. Subsequently, fusion of the autophagosome with the lysosome causes formation of the autolysosome, degradation of its content, and membrane transporter-facilitated release of amino acids and other degradation products into the cytoplasm. Regulation of autophagy is mediated both by stimulatory and inhibitory inputs. Under conditions of sufficient supply with nutrients, in particular amino acids, autophagy is repressed by activation of the inhibitory pathway, amongst others *via* the insulin-mTOR signaling axis. ULK1 (as well as ULK2) forms a complex with two other proteins, the mammalian homologue of the yeast Atg13 (mAtg13) and focal adhesion kinase family-interacting protein of 200 kDa FIP200 (Rb1CC1). During nutrient abundance, ULK1, ULK2 and mAtg13 are directly phosphorylated by mTOR (and also AKT), resulting in ULK1 kinase inhibition and suppression of autophagy. On the other hand, activating AMPK-mediated ULK1 phosphorylation at a different site causes binding of ULK1 to 14-3-3 proteins and subsequent autophagy induction . On the mRNA level, ATF4 activates *ULK1* transcription under conditions of nutrient - namely amino acid - limitation, contributing to stimulated autophagy and release of the nutrients into the cytoplasm .

Mammalian **AMP-activated protein kinase (AMPK)** is activated by ATP depletion which results in an increase of the cellular AMP/ATP ratio. The allosteric AMPK activation is antagonized by ATP. Furthermore, activating AMPK phosphorylation is catalyzed by an upstream kinase, the AMP-activated protein kinase kinase (AMPKK), which is itself activated by AMP. In addition, AMP activates AMPK by making it a better substrate for AMPKK and a worse substrate for protein phosphatases. Therefore, AMPK serves as a cellular fuel gauge that, once activated, switches off a number of ATP-consuming pathways including fatty acid and cholesterol synthesis, resulting in the conservation of cellular energy expenditure. On the other hand, AMPK activation causes an increase in the supply of ATP by enhancing the rate of fatty acid oxidation. In this context, inactivating acetyl-CoA carboxylase phosphorylation that results in decreased malonyl-CoA represents a key step. Furthermore, activated AMPK increases glucose oxidation in skeletal muscle by stimulating glucose transport. In summary, the AMPK cascade plays an essential role in restoring the cellular energy balance in response to an ATP decrease during periods of cellular energy stress . The AMPK holo-enzyme represents a hetero-trimer composed of one catalytic subunit (α) and two regulatory subunits (β and γ). There are two isoforms each of the α and β subunits and three isoforms of the γ subunit. The N-termini of the α subunits contain the catalytic domain and a phosphorylation site for upstream kinases that regulate their activity. The γ subunits are regulatory subunits that bind AMP. The conserved C-termini of the β subunits interact with both the α and the γ subunits and play an obligatory role in AMPK complex formation. In addition, β subunits contain a conserved carbohydrate-binding domain that allows AMPK to function as a glycogen sensor . While AMPK is present in all tissues, the individual AMPK subunits exhibit variation in tissue-specific expression, subunit association, and subcellular localization. Earlier studies demonstrated that the α2 catalytic subunit associates exclusively with β1 in slow-twitch (type I) muscle fibers, whereas in fast-twitch (type II) muscle fibers, α2 associates with both β1 and β2 subunits. The β1 subunit is highly expressed in brain and liver, whereas β2 shows the highest expression in skeletal muscle. In addition, β1 is predominantly located in the nucleus whereas β2 is predominantly cytoplasmic. Subcellular localization of β subunits is of particular importance for AMPK function, as it has been demonstrated that they direct localization of the catalytic α subunit and thus regulate substrate selection.

The **β2** subunit encoded by ***PRKAB2*** plays an important role in regulating glucose, glycogen, and lipid metabolism. Expression of β2 is restricted primarily to brain, heart, kidney, and skeletal muscle, with the latter tissue expressing almost exclusively β2. Skeletal muscle plays a pivotal role in glucose homeostasis. Impaired muscle glucose metabolism induced by high-fat Western diet is frequently associated with metabolic syndrome, and AMPK in general opposes the associated deleterious effects. Glucose metabolism is increased by AMPK through stimulated translocation of glucose transporters to the cell surface. Furthermore, β2 is necessary for exercise-induced AMPK activity as well as of increased transcription of PGC-1α and mitochondrial PGC-1α target genes. Consistently, β2 knockout mice fail to maintain euglycemia and muscle ATP levels during fasting. Furthermore, if maintained on a high-fat diet, they develop high body fat compared to wild-type animals and manifest classic features of metabolic syndrome including hyperglycemia, glucose intolerance, and insulin resistance. Cell surface-associated Glut4 glucose transporter levels are reduced in skeletal muscle of β2 mutant animals on a high-fat diet. In addition, β2 knockout mice are unable to maintain muscle ATP levels during exercise, display poor exercise performance and impaired muscle glycogen metabolism, and show decreased activation of AMPK and deficits in PGC-1α-mediated transcription . In our whole-blood analyses, the amounts of *PRKAB2*-specific mRNA were negatively correlated with BMI. Transferred to skeletal muscle tissue, this finding could be predicted to contribute to impaired glucose metabolism known as a characteristic feature of the metabolic syndrome, which, on the other hand, is epidemiologically strongly associated with increased BMI.

Whereas γ1 and γ2 are widely expressed in human tissues, the expression of **γ3** is essentially restricted to skeletal muscle. The AMP dependence of AMPK is significantly affected by the γ isoform: While γ2-containing enzyme complexes exhibit the strongest AMP-dependence, γ3-containing enzyme complexes are least AMP-dependent and γ1-containing enzyme complexes show intermediate AMP dependence . In our analyses, the ***PRKAG2*** mRNA specifying the γ2 isoform exhibited decreased mRNA amounts correlated with BMI whereas ***PRKAG1*** specifying the γ1 subunit showed increased amounts. These findings suggest that an increased BMI may be associated with a composition of γ subunits that renders the holo-enzyme less AMP-dependent.

The decreased transcript amounts of ***PRKCB*** encoding the **classical** **protein kinase C, beta type (PKCβ)** at increased BMI also tend to improve insulin signaling. Activation of PKC is associated with endothelial dysfunction and insulin resistance in vascular tissue. In an animal model of obesity-associated insulin resistance (Zucker rats), comparison of obese and lean animals revealed increased concentrations of PKC-activating diacylglycerol (DAG), directly measured PKC activity, as well as blunted insulin-stimulated increases in AKT phosphorylation and cGMP concentration as a measure of NO bioavailability in the aorta . Treatment with a PKC inhibitor partly normalized these effects. In cultured endothelial cells, overexpression of PKCβ1 and PKCβ2, but not PKCα, PKCδ, or PKCζ decreased insulin-stimulated AKT phosphorylation and eNOS expression. Insulin-stimulated AKT phosphorylation was furthermore decreased in the microvasculature of transgenic mice overexpressing exclusively PKCβ2 . Thus, PKCβ activation in endothelial cells and vascular tissue inhibits AKT activation by insulin, consequently inhibits AKT-dependent eNOS regulation by insulin, and causes endothelial dysfunction in obesity-associated insulin resistance. Lower expression of *PRKCB* should therefore result in improved insulin signaling.

The **protein tyrosine phosphatase PTP1B** encoded by ***PTPN1*** represents a negative regulator of insulin signaling as it dephosphorylates the activated insulin receptor and the activated IRS proteins, thereby inactivating these proteins . The lower *PTPN1* mRNA amounts at increased BMI detected in our study should therefore improve transduction of the insulin signal, due to attenuated inactivating dephosphorylation.

The same holds true for ***JAK1***: Activation of **c-Jun N-terminal kinases (JNKs)** by pro-inflammatory cytokines and by insulin results in the inhibition of insulin signaling by inhibitory serine phosphorylation of IRS. Thus, JNK kinases serve as heterologous inhibitors of insulin action during inflammation and as feedback inhibitors during insulin stimulation . The lower amounts of ***JAK1*** mRNA at increased BMI should therefore improve transduction of the insulin signal, due to diminished inactivating dephosphorylation.

Phosphorylation of **TSC2** by AKT results in the activation of the mTOR1 pathway. AKT-mediated phosphorylation causes inhibition of the GTPase-stimulating activity of the membrane-associated **TSC1-TSC2 complex**. In the absence of insulin, this complex stimulates the GTPase activity of RHEB (RAS homolog enriched in brain), thereby causing RHEB accumulation in the GDP-bound form that is unable to bind to FKBP38 (FK506 binding protein 8). FKBP38 then binds and inhibits the mTOR1 complex. Insulin-stimulation *via* AKT-dependent inactivating TSC2 phosphorylation mediates the accumulation of RHEB in its active GTP-bound form that binds FKBP38, thereby reversing the inhibition of the mTOR1 kinase complex . The decreased amounts of ***TSC1*** and ***TSC2*** mRNA under conditions of increased BMI should therefore functionally mimic insulin stimulation, as reduced expression of the encoded proteins can be predicted to mirror TSC1-TSC2 inactivation by phosphorylation, finally resulting in mTOR1 activation.

The so-called **forkhead box O (FOXO) transcription factors** are implicated in diverse cellular processes ranging from glucose metabolism to cell behavior including cell cycle and apoptosis . Of special interest, FOXO proteins, in direct interaction with PGC-1α, drive the expression of the gluconeogenic key genes *PCK1* and *G6PC* encoding phosphoenolpyruvate carboxykinase 1 and the catalytic sub-unit of glucose-6-phosphatase, respectively, in liver . In vascular endothelial cells, the management of oxidative stress through up-regulation of antioxidant genes like *SOD2* and *CAT* encoding mitochondrial superoxide dismutase and catalase, respectively, is mediated *via* the interacting transcription factors PGC-1α and FOXO3 . In addition to their role as transcription factors, FOXO proteins also represent co-repressors and co-activators. Consequently, direct DNA binding of FOXOs is not always necessary for the modulation of their target gene expression .

In response to insulin, FOXO transcription factors are phosphorylated by AKT, resulting in their nuclear exclusion and cytoplasmic sequestration. Subsequently, they are poly-ubiquitinated and finally degraded. Among other effects, this ensures down-regulation of gluconeogenesis and protection against oxidative stress as an adaptation to increased glucose availability . In addition to post-translational regulation, FOXO proteins are also regulated at the mRNA level: The transcription of ***FOXO1*** and ***FOXO4*** was found to be stimulated by FOXO3 and possibly by other FOXO factors in a positive feedback loop, which is disrupted by growth factors .

*3. Genes belonging to the major cellular defense system against oxidative stress, the NRF2-regulon*

The nuclear factor E2-related factor 2 (**NRF2**) encoded by ***NFE2L2*** represents the key transcriptional regulator of the major cellular defense system against oxidative stress. It belongs to a basic region leucine zipper (bZip)-type transcription factor family that shares a conserved structural ‘cap n collar’ domain and was originally identified as an erythroid-restricted DNA-binding activity. NRF2 is the most potent transcription factor of this family and activates downstream targets about 100-fold . Under homeostatic conditions, NRF2 is sequestered in the cytoplasm through interaction with the actin-bound protein KEAP1, where this binding serves as an adaptor for the cullin3/ring box 1 (CUL3/RBX1) E3 ubiquitin ligase complex, thereby mediating NRF2 ubiquitination and subsequent degradation of the protein by the 26S proteasome. Under these conditions, NRF2 is an unstable protein with an estimated half-life of about 30 min . Four conserved cysteine residues within the BTB/Kelch repeat region of KEAP1 serve as sensors for inducing ligands or increased cellular oxidative stress. Oxidation of these cysteines by reactive oxygen species or lipid oxidation products causes conformational changes and NRF2 release. Similarly, phosphorylation of NRF2 Ser40 by protein kinase C (PKC) leads to dissociation of the NRF2-KEAP1 complex. In addition, NRF2 activity is also regulated by inhibitory GSK3β-dependent NRF2 phosphorylation. Importantly, stimulation of insulin signaling triggers AKT-mediated inhibitory phosphorylation of GSK3β at Ser9 thereby causing NRF2 activation. It has been demonstrated that GSK3β inhibits NRF2 *via* phosphorylation of ten potential serine residues, causing NRF2 sequestration in the cytoplasm and subsequent degradation. Alternatively, it was described that GSK3β does not directly phosphorylate NRF2 but instead phosphorylates threonine residues of the Src subfamily kinases SRC, YES, FYN, and FGR, which subsequently translocate to the nucleus and in turn directly phosphorylate Tyr568 of NRF2. Finally, this also results in the export, ubiquitination and degradation of NRF2 .

After release from KEAP1, NRF2 translocates to the nucleus where it binds to the *cis*-acting enhancer ARE sequence (core sequence: TGAG/CNNNGC) located in promoters of a battery of genes encoding proteins necessary for glutathione synthesis and electrophile detoxification . Microarray results suggest that more than 200 genes are regulated by NRF2, where one main class of the encoded proteins includes anti-oxidative enzymes like NAD(P)H:quinone oxidoreductase, epoxide hydrolase, aldehyde dehydrogenase, aldoketo reductase, catalase, heme oxygenase-1, and Mn-superoxide dismutase. A second class of proteins encompasses enzymes involved in glutathione homeostasis, including glutathione reductases, peroxiredoxin, thioredoxin and thioredoxin reductases as well as glutathione peroxidase. In addition, NRF2 enhances toxin export through multidrug response transporters, like the multidrug resistance-associated protein, carboxyl esterase, esterase D, retinal oxidase/aldehyde oxidase and carbonic anhydrase. Finally, there are NRF2-regulated genes that encode proteins belonging to different categories, like 26S proteasome subunits and several heat-shock proteins .

Several of these known NRF2 target genes exhibited attenuated expression on the mRNA level under conditions of increased BMI, consistent with a lower nuclear NRF2 activity:

The ***GSTM2*** encoded **glutathione S-transferase~~s~~ µ2** belongs to the cytoplasmic µ class of the glutathione S-transferase (GST) enzyme family and functions in the detoxification of electrophilic compounds, including carcinogens, therapeutic drugs, environmental toxins and products of oxidative stress by catalyzing their conjugation with reduced glutathione .

The ***MGST2***-encoded **microsomal glutathione S-transferase 2** belongs to the MAPEG (Membrane Associated Proteins in Eicosanoid and Glutathione metabolism) protein family and catalyzes the conjugation of leukotriene A4 and reduced glutathione to produce leukotriene C4 .

The ***NQO2***-encoded **NAD(P)H:quinone acceptor oxidoreductase 2** represents an ubiquitously expressed cytosolic FAD-dependent flavoprotein catalyzing obligatory 2-electron reductions of quinone substrates and uses dihydronicotinamide riboside as a reducing coenzyme. As the two-electron reductions depress the quinone levels, they minimize the generation of reactive oxygen intermediates by redox cycling as well as the depletion of intracellular thiol pools. Mutations in this gene have been associated with neurodegenerative diseases and several cancers .

The ***SOD2***-encoded superoxide dismutase 2 represents a homotetrameric mitochondrial matrix enzyme that contains manganese (Mn) as co-factor (**MnSOD**). The Mn at the active site functions in the catalysis of the disproportionation of O_2_^-^ to O_2_ and H_2_O_2_. The enzyme is synthesized in the cytoplasm and directed to the mitochondria by a signal peptide where it is involved in dismutating O_2_^-^ generated as a respiratory chain by-product .

The ***TXNRD1***-encoded thioredoxin reductase 1 represents a member of the family of pyridine nucleotide oxidoreductases. The homodimeric enzyme which contains one selenocysteine (Sec) residue per subunit and uses FAD as a cofactor plays an important role in the protection against oxidative stress by reducing and thus regenerating thioredoxin. The latter is utilized by peroxiredoxins to reduce ROS levels .

The transcriptional activation of ARE-mediated genes requires hetero-dimerization of NRF2 with other basic leucine zipper proteins, including JUN (**c-JUN**, **JUN-D** and **JUN-B**) and the small musculoaponeurotic fibrosarcoma (MAF) proteins (**MAFG**, **MAFK** and **MAFF**). The dimerization strongly activates transcription of downstream targets by enhancing the specificity to bind to a *cis*-acting enhancer of the ARE contained in the promoters of these genes. As the NRF2 encoding gene *NFE2L2* contains two ARE-like sequences in its promoter, NRF2 auto-regulates its extended expression in a positive feedback-loop .

*4. Selected literature concerning micro-RNAs (miRNAs) differentially expressed in insulin signaling- and insulin resistance-relevant tissue (liver, skeletal muscle, adipose tissue, and pancreatic beta cells) during obesity, hyperglycemia, and diabetes.*

References .

**SUPPLEMENTARY REFERENCES**

1. Zambon AC, Gaj S, Ho I, Hanspers K, Vranizan K, Evelo CT, Conklin BR, Pico AR, Salomonis N: **GO-Elite: a flexible solution for pathway and ontology over-representation**. *Bioinformatics* 2012, **28**(16):2209-2210.

2. Cheng Z, Tseng Y, White MF: **Insulin signaling meets mitochondria in metabolism**. *Trends in endocrinology and metabolism: TEM* 2010, **21**(10):589-598.

3. Yang DQ, Halaby MJ, Li Y, Hibma JC, Burn P: **Cytoplasmic ATM protein kinase: an emerging therapeutic target for diabetes, cancer and neuronal degeneration**. *Drug discovery today* 2011, **16**(7-8):332-338.

4. Tabatabaian F, Dougherty K, Di Fulvio M, Gomez-Cambronero J: **Mammalian target of rapamycin (mTOR) and S6 kinase down-regulate phospholipase D2 basal expression and function**. *The Journal of biological chemistry* 2010, **285**(25):18991-19001.

5. Avila-Flores A, Santos T, Rincon E, Merida I: **Modulation of the mammalian target of rapamycin pathway by diacylglycerol kinase-produced phosphatidic acid**. *The Journal of biological chemistry* 2005, **280**(11):10091-10099.

6. Yang KJ, Shin S, Piao L, Shin E, Li Y, Park KA, Byun HS, Won M, Hong J, Kweon GR *et al*: **Regulation of 3-phosphoinositide-dependent protein kinase-1 (PDK1) by Src involves tyrosine phosphorylation of PDK1 and Src homology 2 domain binding**. *The Journal of biological chemistry* 2008, **283**(3):1480-1491.

7. Feller SM: **Crk family adaptors-signalling complex formation and biological roles**. *Oncogene* 2001, **20**(44):6348-6371.

8. Janssens V, Goris J: **Protein phosphatase 2A: a highly regulated family of serine/threonine phosphatases implicated in cell growth and signalling**. *The Biochemical journal* 2001, **353**(Pt 3):417-439.

9. Rodgers JT, Vogel RO, Puigserver P: **Clk2 and B56beta mediate insulin-regulated assembly of the PP2A phosphatase holoenzyme complex on Akt**. *Molecular cell* 2011, **41**(4):471-479.

10. Tsuruzoe K, Emkey R, Kriauciunas KM, Ueki K, Kahn CR: **Insulin receptor substrate 3 (IRS-3) and IRS-4 impair IRS-1- and IRS-2-mediated signaling**. *Molecular and cellular biology* 2001, **21**(1):26-38.

11. Brutman-Barazani T, Horovitz-Fried M, Aga-Mizrachi S, Brand C, Brodie C, Rosa J, Sampson SR: **Protein kinase Cdelta but not PKCalpha is involved in insulin-induced glucose metabolism in hepatocytes**. *Journal of cellular biochemistry* 2012, **113**(6):2064-2076.

12. Standaert ML, Bandyopadhyay G, Perez L, Price D, Galloway L, Poklepovic A, Sajan MP, Cenni V, Sirri A, Moscat J *et al*: **Insulin activates protein kinases C-zeta and C-lambda by an autophosphorylation-dependent mechanism and stimulates their translocation to GLUT4 vesicles and other membrane fractions in rat adipocytes**. *The Journal of biological chemistry* 1999, **274**(36):25308-25316.

13. Romanelli A, Martin KA, Toker A, Blenis J: **p70 S6 kinase is regulated by protein kinase Czeta and participates in a phosphoinositide 3-kinase-regulated signalling complex**. *Molecular and cellular biology* 1999, **19**(4):2921-2928.

14. Hirai T, Chida K: **Protein kinase Czeta (PKCzeta): activation mechanisms and cellular functions**. *Journal of biochemistry* 2003, **133**(1):1-7.

15. Hu J, Liu J, Ghirlando R, Saltiel AR, Hubbard SR: **Structural basis for recruitment of the adaptor protein APS to the activated insulin receptor**. *Molecular cell* 2003, **12**(6):1379-1389.

16. Lang F, Gorlach A, Vallon V: **Targeting SGK1 in diabetes**. *Expert opinion on therapeutic targets* 2009, **13**(11):1303-1311.

17. Brady MJ, Saltiel AR: **The role of protein phosphatase-1 in insulin action**. *Recent progress in hormone research* 2001, **56**:157-173.

18. Herzig S, Long F, Jhala US, Hedrick S, Quinn R, Bauer A, Rudolph D, Schutz G, Yoon C, Puigserver P *et al*: **CREB regulates hepatic gluconeogenesis through the coactivator PGC-1**. *Nature* 2001, **413**(6852):179-183.

19. Houten SM, Auwerx J: **PGC-1alpha: turbocharging mitochondria**. *Cell* 2004, **119**(1):5-7.

20. Papa S, Rasmo DD, Technikova-Dobrova Z, Panelli D, Signorile A, Scacco S, Petruzzella V, Papa F, Palmisano G, Gnoni A *et al*: **Respiratory chain complex I, a main regulatory target of the cAMP/PKA pathway is defective in different human diseases**. *FEBS letters* 2012, **586**(5):568-577.

21. Zmuda-Trzebiatowska E, Oknianska A, Manganiello V, Degerman E: **Role of PDE3B in insulin-induced glucose uptake, GLUT-4 translocation and lipogenesis in primary rat adipocytes**. *Cellular signalling* 2006, **18**(3):382-390.

22. Degerman E, Ahmad F, Chung YW, Guirguis E, Omar B, Stenson L, Manganiello V: **From PDE3B to the regulation of energy homeostasis**. *Current opinion in pharmacology* 2011, **11**(6):676-682.

23. Qi L, Saberi M, Zmuda E, Wang Y, Altarejos J, Zhang X, Dentin R, Hedrick S, Bandyopadhyay G, Hai T *et al*: **Adipocyte CREB promotes insulin resistance in obesity**. *Cell metabolism* 2009, **9**(3):277-286.

24. Jones KW, Shapero MH, Chevrette M, Fournier RE: **Subtractive hybridization cloning of a tissue-specific extinguisher: TSE1 encodes a regulatory subunit of protein kinase A**. *Cell* 1991, **66**(5):861-872.

25. Regazzetti C, Bost F, Le Marchand-Brustel Y, Tanti JF, Giorgetti-Peraldi S: **Insulin induces REDD1 expression through hypoxia-inducible factor 1 activation in adipocytes**. *The Journal of biological chemistry* 2010, **285**(8):5157-5164.

26. Reddy NM, Kleeberger SR, Yamamoto M, Kensler TW, Scollick C, Biswal S, Reddy SP: **Genetic dissection of the Nrf2-dependent redox signaling-regulated transcriptional programs of cell proliferation and cytoprotection**. *Physiological genomics* 2007, **32**(1):74-81.

27. Zelzer E, Levy Y, Kahana C, Shilo BZ, Rubinstein M, Cohen B: **Insulin induces transcription of target genes through the hypoxia-inducible factor HIF-1alpha/ARNT**. *The EMBO journal* 1998, **17**(17):5085-5094.

28. Pike LR, Singleton DC, Buffa F, Abramczyk O, Phadwal K, Li JL, Simon AK, Murray JT, Harris AL: **Transcriptional up-regulation of ULK1 by ATF4 contributes to cancer cell survival**. *The Biochemical journal* 2013, **449**(2):389-400.

29. Bach M, Larance M, James DE, Ramm G: **The serine/threonine kinase ULK1 is a target of multiple phosphorylation events**. *The Biochemical journal* 2011, **440**(2):283-291.

30. Cheung PC, Salt IP, Davies SP, Hardie DG, Carling D: **Characterization of AMP-activated protein kinase gamma-subunit isoforms and their role in AMP binding**. *The Biochemical journal* 2000, **346 Pt 3**:659-669.

31. Dasgupta B, Ju JS, Sasaki Y, Liu X, Jung SR, Higashida K, Lindquist D, Milbrandt J: **The AMPK beta2 subunit is required for energy homeostasis during metabolic stress**. *Molecular and cellular biology* 2012, **32**(14):2837-2848.

32. Naruse K, Rask-Madsen C, Takahara N, Ha SW, Suzuma K, Way KJ, Jacobs JR, Clermont AC, Ueki K, Ohshiro Y *et al*: **Activation of vascular protein kinase C-beta inhibits Akt-dependent endothelial nitric oxide synthase function in obesity-associated insulin resistance**. *Diabetes* 2006, **55**(3):691-698.

33. Tiganis T: **PTP1B and TCPTP--nonredundant phosphatases in insulin signaling and glucose homeostasis**. *The FEBS journal* 2013, **280**(2):445-458.

34. Lee YH, Giraud J, Davis RJ, White MF: **c-Jun N-terminal kinase (JNK) mediates feedback inhibition of the insulin signaling cascade**. *The Journal of biological chemistry* 2003, **278**(5):2896-2902.

35. Ponugoti B, Dong G, Graves DT: **Role of forkhead transcription factors in diabetes-induced oxidative stress**. *Experimental diabetes research* 2012, **2012**:939751.

36. Puigserver P, Rhee J, Donovan J, Walkey CJ, Yoon JC, Oriente F, Kitamura Y, Altomonte J, Dong H, Accili D *et al*: **Insulin-regulated hepatic gluconeogenesis through FOXO1-PGC-1alpha interaction**. *Nature* 2003, **423**(6939):550-555.

37. Olmos Y, Valle I, Borniquel S, Tierrez A, Soria E, Lamas S, Monsalve M: **Mutual dependence of Foxo3a and PGC-1alpha in the induction of oxidative stress genes**. *The Journal of biological chemistry* 2009, **284**(21):14476-14484.

38. Essaghir A, Dif N, Marbehant CY, Coffer PJ, Demoulin JB: **The transcription of FOXO genes is stimulated by FOXO3 and repressed by growth factors**. *The Journal of biological chemistry* 2009, **284**(16):10334-10342.

39. Martin-Montalvo A, Villalba JM, Navas P, de Cabo R: **NRF2, cancer and calorie restriction**. *Oncogene* 2011, **30**(5):505-520.

40. Vomhof-Dekrey EE, Picklo MJ, Sr.: **The Nrf2-antioxidant response element pathway: a target for regulating energy metabolism**. *The Journal of nutritional biochemistry* 2012, **23**(10):1201-1206.

41. Andonova IE, Justenhoven C, Winter S, Hamann U, Baisch C, Rabstein S, Spickenheuer A, Harth V, Pesch B, Bruning T *et al*: **No evidence for glutathione S-transferases GSTA2, GSTM2, GSTO1, GSTO2, and GSTZ1 in breast cancer risk**. *Breast cancer research and treatment* 2010, **121**(2):497-502.

42. Ikeda H, Serria MS, Kakizaki I, Hatayama I, Satoh K, Tsuchida S, Muramatsu M, Nishi S, Sakai M: **Activation of mouse Pi-class glutathione S-transferase gene by Nrf2(NF-E2-related factor 2) and androgen**. *The Biochemical journal* 2002, **364**(Pt 2):563-570.

43. Wang W, Jaiswal AK: **Nuclear factor Nrf2 and antioxidant response element regulate NRH:quinone oxidoreductase 2 (NQO2) gene expression and antioxidant induction**. *Free radical biology & medicine* 2006, **40**(7):1119-1130.

44. Fukai T, Ushio-Fukai M: **Superoxide dismutases: role in redox signaling, vascular function, and diseases**. *Antioxidants & redox signaling* 2011, **15**(6):1583-1606.

45. Yu ZW, Li D, Ling WH, Jin TR: **Role of nuclear factor (erythroid-derived 2)-like 2 in metabolic homeostasis and insulin action: A novel opportunity for diabetes treatment?** *World journal of diabetes* 2012, **3**(1):19-28.

46. Gorrini C, Harris IS, Mak TW: **Modulation of oxidative stress as an anticancer strategy**. *Nature reviews Drug discovery* 2013, **12**(12):931-947.

47. He A, Zhu L, Gupta N, Chang Y, Fang F: **Overexpression of micro ribonucleic acid 29, highly up-regulated in diabetic rats, leads to insulin resistance in 3T3-L1 adipocytes**. *Molecular endocrinology* 2007, **21**(11):2785-2794.

48. Williams MD, Mitchell GM: **MicroRNAs in insulin resistance and obesity**. *Experimental diabetes research* 2012, **2012**:484696.

**LEGEND FOR SUPPLEMENTARY FIGURE**

**Correlation of Signature Transcripts with BMI-related Traits.** Dark red, significantly positive associations, light red, nominally (p < 0.05) positive associations, dark green, significantly negative association, light green, nominally negative associations. The significance threshold was set to a level corresponding to a Benjamini-Hochberg p-value < 0.01 in the TWAS on BMI.
