# Supplementary figures and images for: Extensive alterations of the whole-blood transcriptome are associated with body mass index: results of an mRNA profiling study involving two large population-based cohorts
Source: BMC Med Genomics. 2015 Oct 15;8:65. doi: 10.1186/s12920-015-0141-x (PMC4608219; doi:10.1186/s12920-015-0141-x)

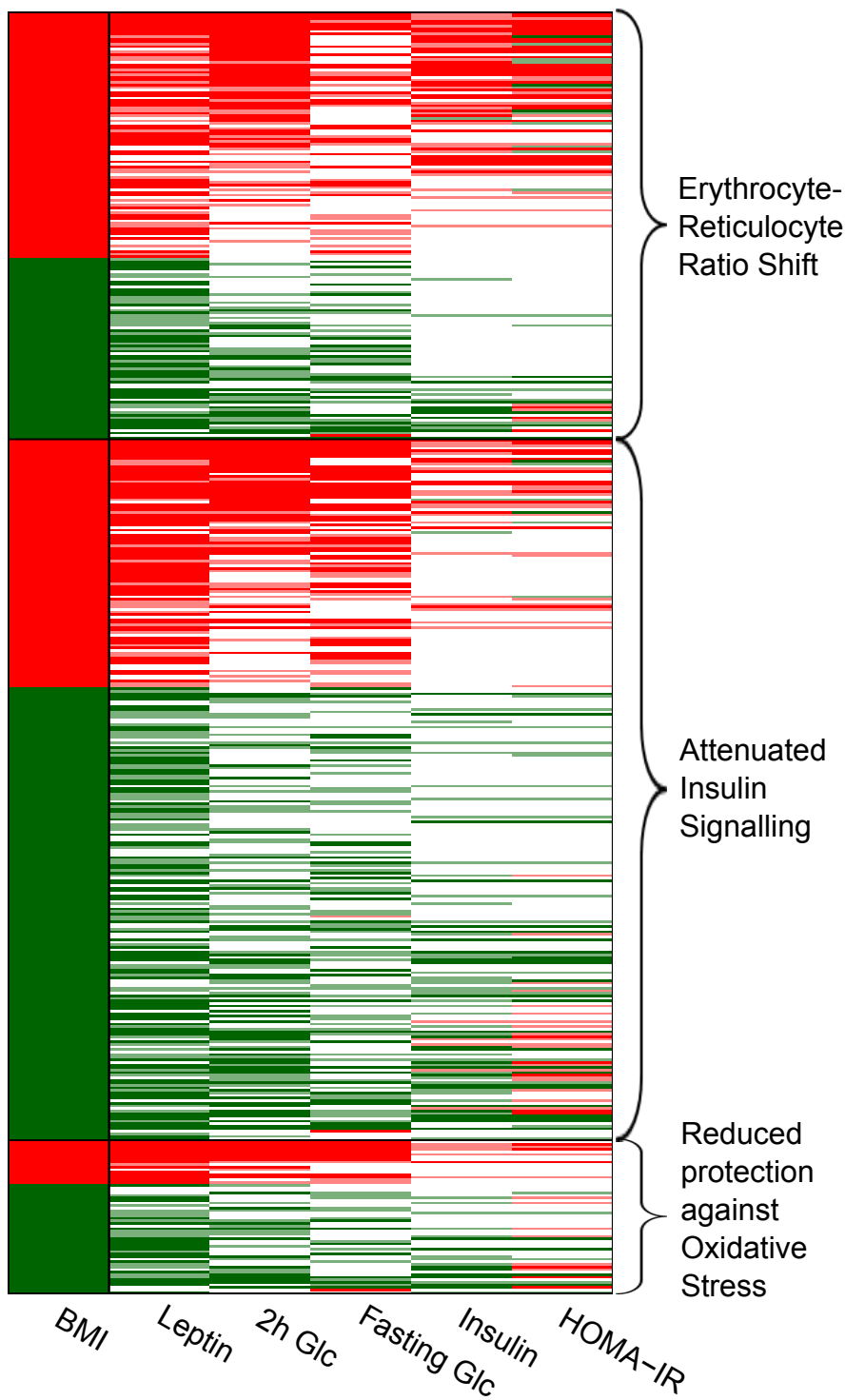

Supplement: Supplementary file 5 — Correlation of Signature Transcripts with BMI-related Traits. (PDF 39 kb) [file 12920_2015_141_MOESM5_ESM.pdf]
